# Domain III *β*4–*β*5 Loop and *β*14–*β*15 Loop of *Bacillus thuringiensis* Vip3Aa Are Involved in Receptor Binding and Toxicity

**DOI:** 10.3390/toxins16010023

**Published:** 2024-01-01

**Authors:** Xiaoyue Hou, Mengjiao Li, Chengjuan Mao, Lei Jiang, Wen Zhang, Mengying Li, Xiaomeng Geng, Xin Li, Shu Liu, Guang Yang, Jing Zhou, Yaowei Fang, Jun Cai

**Affiliations:** 1 Jiangsu Key Laboratory of Marine Bioresources and Environment, Jiangsu Ocean University, Lianyungang 222005, China; 2020000057@jou.edu.cn (X.H.); 2007000028@jou.edu.cn (S.L.); 2020000050@jou.edu.cn (G.Y.); 2 Co–Innovation Center of Jiangsu Marine Bio–Industry Technology, Jiangsu Ocean University, Lianyungang 222005, China; 3 Jiangsu Marine Resources Development Research Institute, Jiangsu Ocean University, Lianyungang 222005, China; 4 College of Marine Food and Bioengineering, Jiangsu Ocean University, Lianyungang 222005, China; xydxhl@163.com (M.L.); mcj15055202109@163.com (C.M.); jl17892521856@163.com (L.J.); zw19975066385@163.com (W.Z.); lmy13775446376@163.com (M.L.); gxm18913965276@163.com (X.G.); 2021122077@jou.edu.cn (X.L.); 5 College of Life Sciences, Nankai University, Tianjin 300071, China; 6 Lianyungang City Quality Technology Comprehensive Inspection and Quality Inspection Center, Lianyungang 222346, China; zj13179573851@163.com

**Keywords:** microbial pesticides, Vip3Aa, domain III, binding affinity, *Spodoptera frugiperda*

## Abstract

Vip3Aa, secreted by *Bacillus thuringiensis*, is effective at controlling major agricultural pests such as *Spodoptera frugiperda.* However, to control Vip3Aa resistance evolved in the field by different lepidoptera species, an in–depth study of sequence––structure––activity relationships is necessary to design new Vip3Aa variants. In this study, the four specific loops (*β*4–*β*5 loop, *β*9–*β*10 loop, *β*12–*β*13 loop, and *β*14–*β*15 loop) in domain III were selected and four loop mutants were constructed by replacing all residues in each specific loop with alanine. We obtained soluble proteins for three of the loop mutants, excluding the *β*9–*β*10 loop. These loop mutants have been characterized by toxicity bioassays against *S. frugiperda*, proteolytic processing, and receptor binding. These results indicate that the *β*4–*β*5 loop and *β*14–*β*15 loop are involved in receptor binding and Vip3Aa toxicity. Based on this, we constructed numerous mutants and obtained three single mutants (Vip3Aa–S366T, Vip3Aa–S366L, and Vip3Aa–R501A) that exhibited significantly increased toxicity of 2.61–fold, 3.39–fold, and 2.51–fold, respectively. Compared to Vip3Aa, the receptor affinity of Vip3Aa–S366T and Vip3Aa–S366L was significantly enhanced. Furthermore, we also analyzed and aligned the three–dimensional structures of the mutants and Vip3Aa. In summary, these results indicate that the loops in domain III have the potential to be targeted to enhance the insecticidal toxicity of the Vip3Aa protein.

## 1. Introduction

*Spodoptera frugiperda* (Lepidoptera, Noctuidae) is native to the tropics and subtropics of the Americas and has become a major global invasive pest in the past decade [1]. Planting crops that express insecticidal proteins is a green pest control strategy, as these crops exhibit specific toxicity to different pests [2]. Currently, widely used insecticidal proteins are generally derived from *Bacillus thuringiensis* and are categorized into insecticidal crystal proteins (ICPs) and vegetative insecticidal proteins (Vips). Given that some lepidopteran pests, such as *S. frugiperda*, have developed resistance to certain ICPs, it has become urgent to study Vips that do not share any amino acid sequence homology with ICPs [3].

Based on the protein structure, Vips are divided into three categories: Vip3, Vpa, and Vpb [4]. Currently, the focus of research and application is mainly on Vip3 proteins, which can not only specifically control lepidopteran pests such as *S. frugiperda*, *Agrotis ipsilon*, and *Spodoptera exigua* but are also different from ICPs in terms of their receptor binding sites [5]. The amino acid similarity among Vip3Aa protein members is up to 95% and the average molecular weight is approximately 88 kDa. Vip3A can be hydrolyzed by trypsin or insect midgut juice into two fragments, one of 22 kDa (corresponding to the first 198 amino acids) and the other of 66 kDa (corresponding to the amino acids after position 198). The 66 kDa band was initially believed to be a fragment of toxic activation. With the in–depth study of the mechanism of action of Vip3Aa protein, researchers discovered that after being cleaved by trypsin, the 22 kDa fragment and the 66 kDa fragment still remain bound together, forming the activated toxin [6,7].

Trypsin treatment of alanine mutants revealed that the Vip3Af protein consists of five domains, of which domains I to III are required for protein tetramerization [8]. Domain I consists of highly curved *α*–helices and may be responsible for the ability of the Vip3A protein to insert into the cell membrane; domain I also plays a key role in maintaining the stability of Vip3Aa in the presence of midgut proteases [9,10]. In addition, domains II and III of the Vip3Aa protein are the core regions responsible for its binding with the brush border membrane vesicles (BBMVs) of *S. frugiperda*, with domain II potentially playing a role in stabilizing Vip3 oligomers [7,10]. Domain III was first shown to play a major role in the binding between Vip3Aa and Sf9 cells and recent studies have shown that Vip3Aa truncated variants lacking domain II and domain III or containing only one of them, fail to bind to target cells, suggesting that domain II and domain III together are the receptor–binding domains of the Vip3Aa protein [10,11]. The Vip3 proteins contain carbohydrate–binding modules in domains IV and V [7]. These modules potentially interact with the peritrophic membrane to facilitate the passage of the Vip3 protein through it, enabling contact with the insect midgut epithelium [10].

After determining the 3D structure of the Vip3 protein, there has been a gradual increase in the number of studies investigating its structure––effect relationship. Trypsin cleaves the Vip3Aa protein from its protoxin form to an activated toxin, which is a prerequisite for the insecticidal activity of the Vip3Aa protein. Helix *α*1 plays an important role in restricting the conformation of domain I in the Vip3Aa protoxin and ensuring the insecticidal activity of the Vip3Aa activated toxin [12]. There are multiple protease cleavage sites in the loop region between domain I and domain II and it is advantageous to increase the insecticidal activity of Vip3Aa by adding more cleavage sites [13]. In the 3D protein structure, loops connecting secondary structures play crucial roles in the function of the protein. Domain III contains three antiparallel *β*–sheets and is similar to domain II of Cry3A [7,14]. Domain II of the Cry proteins has receptor recognition and receptor binding functions, in which the loops play an important role [15,16]. Therefore, loops in domain III, especially those exposed on the protein structure surface, may be involved in the binding process between the Vip3Aa protein and its receptor. However, studies on the loops in domain III of the Vip3Aa protein are not yet available, which limits studies related to Vip3Aa protein receptor recognition and receptor binding.

In the present study, we focused on four specific loops (*β*4–*β*5 loop, *β*9–*β*10 loop, *β*12–*β*13 loop, and *β*14–*β*15 loop) in domain III of Vip3Aa and replaced all residues in each of the loops with alanine, respectively, to generate loop alanine mutants. Compared to the wild–type protein Vip3Aa, these mutants exhibited decreased insecticidal activity against *S. frugiperda*, possibly due to reduced stability in the midgut juice or impaired binding to receptors on midgut epithelial cells. In addition, we constructed single mutants and multiple mutants in the *β*4–*β*5 loop and *β*14–*β*15 loop of domain III and found that the mutants with improved insecticidal activity exhibited stronger binding ability to BBMVs of *S. frugiperda*. Finally, we analyzed the potential roles of the *β*4–*β*5 loop and *β*14–*β*15 loop in the insecticidal activity of Vip3Aa using the predicted 3D protein structure. In summary, these results demonstrate that the loops in domain III are involved in receptor binding and toxicity.

## 2. Results

### 2.1. Substituting the Sequence of the Loop in Domain III Reduces the Insecticidal Activity of Vip3Aa

**Figure 1:**
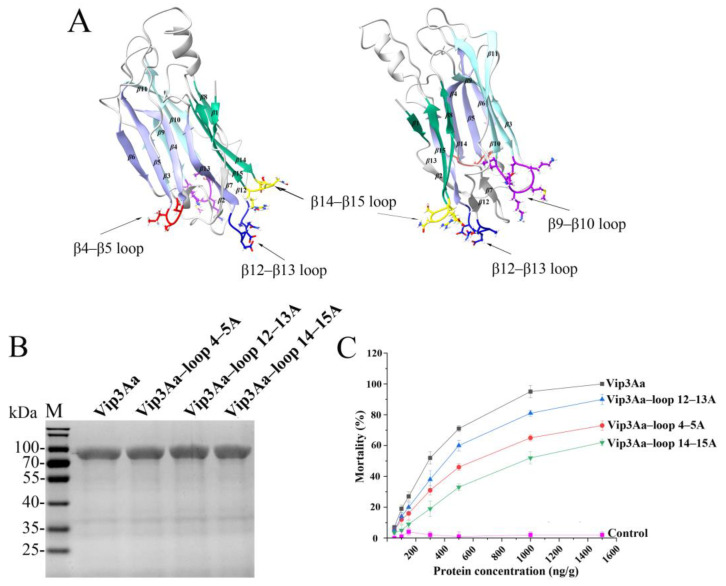
The Vip3Aa loop mutants showed a decrease in toxicity against *S. frugiperda* neonate larvae. (**A**) The spatial location of the four special loops in domain III. (**B**) SDS–PAGE results of Vip3Aa and its mutants. (**C**) The insecticidal activity of Vip3Aa and its mutants.

Vip3Aa contains five domains and domain III, composed of three antiparallel *β*–sheets, plays an important role in the interactions between Vip3Aa and the receptors in *S. frugiperda* BBMVs [8,10]. Four special loops (*β*4–*β*5 loop, *β*9–*β*10 loop, *β*12–*β*13 loop, and *β*14–*β*15 loop) in domain III were selected and mutated in this study to further elucidate the insecticidal mechanism of Vip3Aa (Figure 1A). The *β*4–*β*5 loop (D^365^SI^367^), *β*9–*β*10 loop (K^429^KMKTL^434^), and *β*14–*β*15 loop (E^498^NSR^501^) are from different *β*–sheets in domain III and are protruding loops. The *β*12–*β*13 loop (S^468^ANDDG^473^) is the least conserved loop in Vip3 protein domain III (Figure S1). Moreover, these four loops are exposed to the surface of the Vip3Aa protein (Figure S2). Four loop mutants (Vip3Aa–loop4–5A, Vip3Aa–loop9–10A, Vip3Aa–loop12–13A, and Vip3Aa–loop14–15A) were constructed by replacing all residues in each specific loop with alanine.

Unfortunately, we did not obtain the soluble protein for mutant Vip3Aa–loop9–10A (mutation of residues 428–KKMKTL–435 to 428–AAAAAA–435). As shown in Figure 1B, we obtained mutant proteins with a molecular mass of approximately 88 kDa. These mutant proteins exhibited expression levels similar to those of Vip3Aa. To explore the effect of the mutations in domain III loops, the three–loop residue substitution mutants were bioassayed against neonate larvae of the insect species *S. frugiperda*. The mortality rate of *S. frugiperda* reached 95% when treated with 1000 ng/g Vip3Aa but the lethality rates for Vip3Aa–loop4–5A, Vip3Aa–loop12–13A, and Vip3Aa–loop14–15A at the same concentration were 65%, 81%, and 52%, respectively (Figure 1C). The wild–type protein Vip3Aa showed an LC_50_ value of 251 (226–307) ng/g; however, the mutant proteins Vip3Aa–loop4–5A, Vip3Aa–loop12–13A, and Vip3Aa–loop14–15A showed decreased toxicity compared to Vip3Aa (Table 1).

### 2.2. Substituting the Sequence of the Loop in Domain III Affects the Stability of Vip3Aa

**Figure 2:**
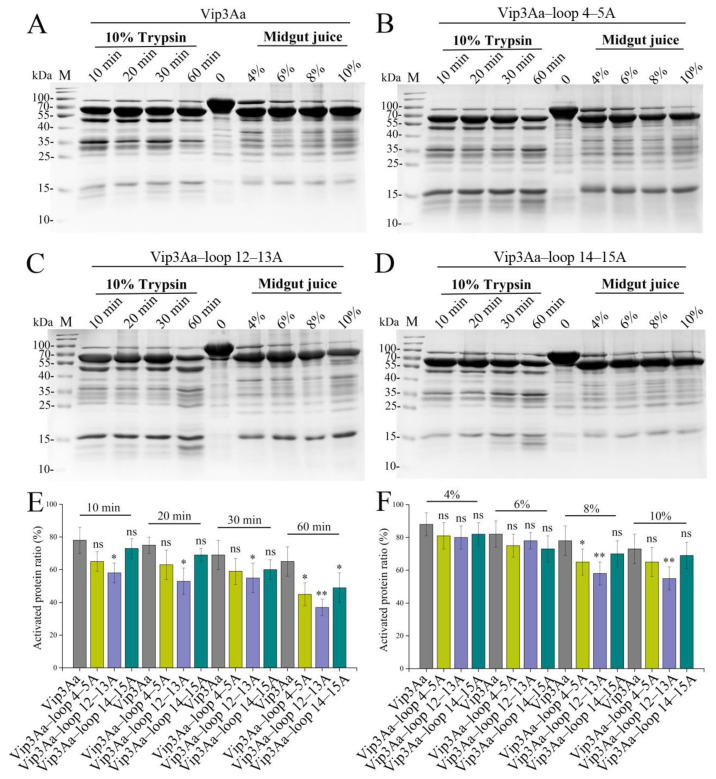
Proteolytic processing of Vip3Aa (**A**), Vip3Aa–loop 4–5A (**B**), Vip3Aa–loop 12–13A (**C**), and Vip3Aa–loop 14–15A (**D**). The cleaved fragments were separated via SDS–PAGE. (**E**) Percentage of 66 kDa fragment after trypsin treatment in Vip3Aa and its mutants. (**F**) Percentage of 66 kDa fragment after *S. frugiperda* midgut juice treatment in Vip3Aa and its mutants. Significant differences from the controls are shown as * *p* < 0.05 and ** *p* < 0.01. ns: no significant.

Domain III is essential to the tetramerization of Vip3 [7,9]. To test the effect of the domain III loop mutation on the proteolytic pattern of Vip3Aa, trypsin and *S. frugiperda* midgut juice were used to treat the mutant proteins. As shown in Figure 2A–D, the mutant proteins and Vip3Aa showed similar proteolytic patterns, with the major fragment being approximately 66 kDa. With the prolongation of trypsin hydrolysis time or the increase in midgut juice concentration, the proteolytic patterns did not change. However, compared to Vip3Aa, the activated band ratio of mutant proteins decreased to varying degrees, especially Vip3Aa–loop12–13A (Figure 2E,F). When the mutant proteins were treated with trypsin for 60 min, there was a significant decrease in the percentage of 66 kDa fragments in all three mutant proteins. Combined with the results of the mutant proteins treated with *S. frugiperda* midgut juice, we conclude that the *β*12–*β*13 loop may have a greater effect on the stability of the Vip3Aa protein than the *β*4–*β*5 loop and *β*14–*β*15 loop.

### 2.3. Substituting the Sequence of the Loop in Domain III Affects the Binding of Vip3Aa to S. frugiperda BBMVs

**Figure 3:**
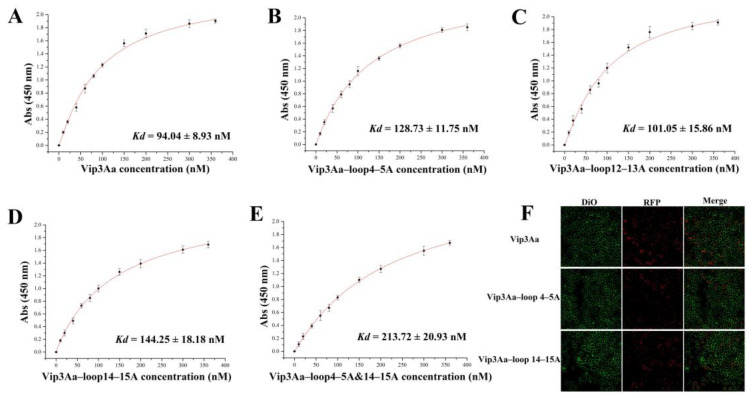
Binding analysis of Vip3Aa and its mutants to *S. frugiperda* BBMVs. Saturation binding of biotinylated Vip3Aa (**A**), Vip3Aa–loop 4–5A (**B**), Vip3Aa–loop 12–13A (**C**), or Vip3Aa–loop 14–15A (**D**) to *S. frugiperda* BBMVs. (**E**) Saturation binding of biotinylated Vip3Aa–loop4–5A&14–15A. (**F**) Confocal microscopy analysis of the entry of Vip3Aa or its mutants into Sf9 cells.

The interaction between Vip3Aa and the target pest midgut BBMVs is widely perceived as the vital step for insecticidal activity [17,18]. To analyze the effect of domain III loop residue substitution with alanine on binding between Vip3Aa and its receptors, we first performed ELISA binding saturation assays of the toxins with biotin tag to *S. frugiperda* BBMVs. As shown in Figure 3A–D, unlike Vip3Aa–loop 12–13A, the equilibrium dissociation constants (*K*_d_) of Vip3Aa–loop 4–5A (128.73 ± 11.75 nM) or Vip3Aa–loop 14–15A (144.25 ± 18.18 nM) binding to BBMVs were significantly larger than that of Vip3Aa (94.04 ± 8.93 nM). Furthermore, when the residues in both *β*4–*β*5 loop and *β*14–*β*15 loop were replaced by alanine, the equilibrium dissociation constant of Vip3Aa–loop4–5A&14–15A binding to BBMVs was elevated even more (Figure 3E). In addition, it was shown that Vip3Aa enters Sf9 cells via receptor–mediated endocytosis and that the amount of Vip3Aa entering the cells correlates with cytotoxicity [19]. To investigate whether the *β*4–*β*5 loop and *β*14–*β*15 loop in domain III are involved in the binding of Vip3Aa to its internalization–related receptors, we constructed the fusion protein composed of Vip3Aa (or Vip3Aa–loop 4–5A or Vip3Aa–loop 14–15A) and red fluorescence protein (RFP). A greater number of red fluorescent dots could be observed in Vip3Aa–RFP–treated cells than in Vip3Aa–loop 4–5A–RFP or Vip3Aa–loop 14–15A–RPF–treated cells; Vip3Aa–loop 4–5A and Vip3Aa–loop 14–15A were also less cytotoxic than Vip3Aa (Figure 3F and Figure S3). These results suggest that the *β*4–*β*5 loop or *β*14–*β*15 loop in domain III should be involved in the interaction between Vip3Aa and its receptors.

### 2.4. Selective Modification of the Loops in Domain III Contributes to the Insecticidal Activity of Vip3Aa

**Figure 4:**
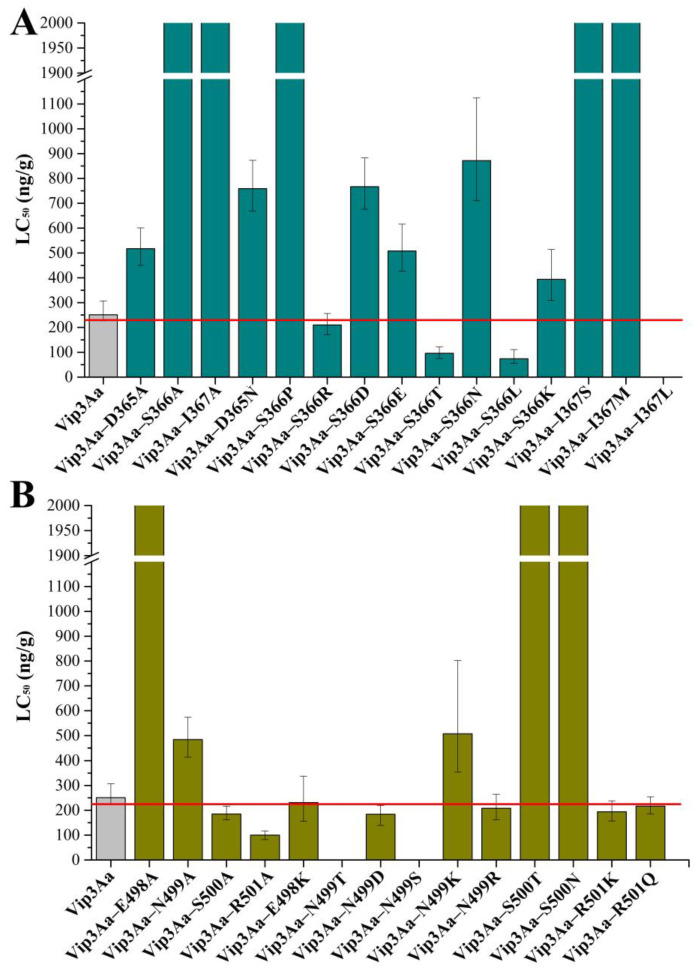
Toxicity of Vip3Aa single mutants against *S. frugiperda* neonate larvae. (**A**) Mutants in the *β*4–*β*5 loop (D^365^SI^367^); (**B**) Mutants in the *β*14–*β*15 loop (E^498^NSR^501^). Vip3Aa–I367L, Vip3Aa–N499T, and Vip3Aa–N499S did not obtain soluble protein.

To better understand the role of residues in the *β*4–*β*5 loop and *β*14–*β*15 loop in the insecticidal activity of Vip3Aa, these residues were modified by site–directed mutagenesis. As indicated in Figure 4A, the insecticidal activity against *S. frugiperda* was significantly impaired when the three residues (D365, S366, and I367) in the *β*4–*β*5 loop were replaced by alanine, especially S366 and I367. Unlike the mutants of the *β*4–*β*5 loop, the mutants of the *β*14–*β*15 loop showed widely varying toxicity. The toxicity of mutant Vip3Aa–E498A was significantly impaired, while the mutants Vip3Aa–S500A (LC50: 185 (162–217) ng/g) and Vip3Aa–R501A (LC50: 100 (82–117) ng/g) showed enhanced toxicity compared to the Vip3Aa protein (LC50: 251 (226–307) ng/g).

In addition, the residues in the *β*4–*β*5 loop and *β*14–*β*15 loop were also substituted with amino acids that differed in the multiple sequence alignments of the Vip3 proteins (Figure S1). However, only five (Vip3Aa–S366R, Vip3Aa–E498K, Vip3Aa–N499R, Vip3Aa–R501K, and Vip3Aa–R501Q) of the 14 mutants displayed toxicity similar to that of the Vip3Aa protein. The remaining 10 mutants either had severely impaired insecticidal toxicity or were unable to be obtained as soluble proteins.

Studies have shown that the mutants (S9N, S193T, and S194L) displayed an approximately two–fold insecticidal activity against *Helicoverpa armigera* larvae compared with Vip3Aa11 [20]. To obtain mutant proteins with enhanced insecticidal activity against *S. frugiperda,* more mutants were generated by replacing serine at position 366 with asparagine, leucine, threonine, and charged polar amino acids (aspartic acid, glutamic acid, and lysine), respectively (Figure 4). The LC_50_ values of Vip3Aa–S366T and Vip3Aa–S366L were 96 (75–122) and 74 (56–111) ng/g, respectively, indicating that these two mutants had approximately 2.6– and 3.4–fold higher activity against *S. frugiperda* than Vip3Aa. However, the insecticidal activity of other mutants was lower than that of Vip3Aa, especially Vip3Aa–S500N and Vip3Aa–S500T (Figure 4A).

The replacement of glutamic acid (E, negative) with lysine (K, positive) at position 498 had little influence on the insecticidal activity of Vip3Aa against *S. frugiperda* (Figure 4B). In a subunit of the Vip3Aa protein tetramer, residue N499 does not have any contact with other residues (Table S2). Based on the difference in hydrophilicity of the amino acids, asparagine at position 499 was substituted with aspartic acid and threonine to obtain mutants Vip3Aa–N499D and Vip3Aa–N499T, respectively. We did not obtain soluble protein for Vip3Aa–N499T. However, Vip3Aa–N499D (LC_50_: 184 (139–219) ng/g) showed slightly increased insecticidal activity over Vip3Aa (LC_50_: 251 (226–307) ng/g).

Thus far, we have obtained five single mutants (Vip3Aa–S366L, Vip3Aa–S366T, Vip3Aa–R501A, Vip3Aa–S500A, and Vip3Aa–N499D) with enhanced insecticidal activity compared to Vip3Aa but Vip3Aa–S500A and Vip3Aa–N499D had less toxicity than the other three single mutants. In addition, compared to Vip3Aa–loop4–5A and Vip3Aa–loop14–15A, the mutant Vip3Aa–loop12–13A showed less impaired insecticidal activity, so we constructed only one mutant (Vip3Aa–N470K) in the *β*12–*β*13 loop, which replaced the asparagine at position 470 with lysine. Mutant Vip3Aa–N470K (LC_50_: 123 (107–141) ng/g) showed enhanced toxicity compared to Vip3Aa (Table 2). Subsequently, multiple mutants were constructed, including Vip3Aa–S366T/R501A, Vip3Aa–S366L/R501A, Vip3Aa–S366T/N470K, Vip3Aa–S366L/N470K, Vip3Aa–N470K/R501A, Vip3Aa–S366T/N470K/R501A, and Vip3Aa–S366L/N470K/R501A. Among these multiple mutants, only Vip3Aa–S366L/R501A did not show enhanced toxicity compared to Vip3Aa. However, only the multiple mutants Vip3Aa–S366T/N470K and Vip3Aa–S366L/N470K showed higher toxicity than Vip3Aa–S366L (Table 2 and Figure 4A). These results suggest that selective modification of loops in domain III can increase the toxicity of Vip3Aa.

### 2.5. Toxicity–Enhanced Vip3Aa Mutants Bind More Strongly to S. frugiperda BBMVs

**Figure 5:**
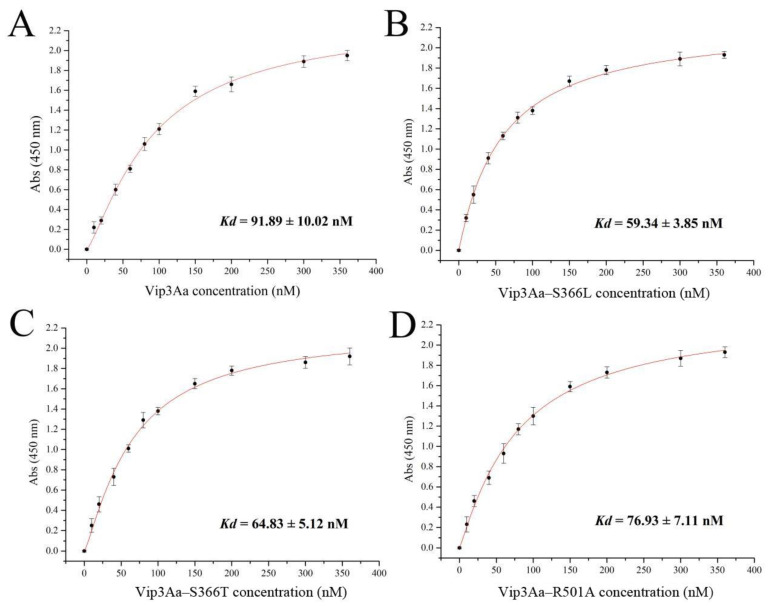
Saturation binding of biotinylated Vip3Aa (**A**), Vip3Aa–S366L (**B**), Vip3Aa–S366T (**C**), or Vip3Aa–R501A (**D**) to *S. frugiperda* BBMVs.

Our results suggested that the *β*4–*β*5 loop or *β*14–*β*15 loop in domain III is involved in the interaction between Vip3Aa and its receptors. Therefore, the binding of toxicity–enhanced Vip3Aa mutants to *S. frugiperda* BBMVs was measured by ELISA binding saturation assays. As shown in Figure 5, the equilibrium dissociation constants (*K*_d_) of Vip3Aa binding to BBMVs, Vip3Aa–S366L, Vip3Aa–S366T, and Vip3Aa–R501A were 91.89 ± 10.02 nM, 59.34 ± 3.85 nM, 64.83 ± 5.12 nM, and 76.93 ± 7.11 nM, respectively. These results indicate that the toxicity–enhanced Vip3Aa mutants (except Vip3Aa–R501A) have greater binding with *S. frugiperda* BBMVs than Vip3Aa.

### 2.6. Three–Dimensional Structural Analysis of Vip3Aa Mutants

Based on the results of the insecticidal activity of the mutants against *S. frugiperda* neonate larvae, we categorized the single mutants into four groups: (i) the insecticidal activity was significantly improved; (ii) the insecticidal activity was severely impaired; (iii) the soluble protein was not available; and (iv) the insecticidal activity was affected. We used the Phyre2 server to predict the 3D structure of the mutants except for mutant (iv).

**Figure 6:**
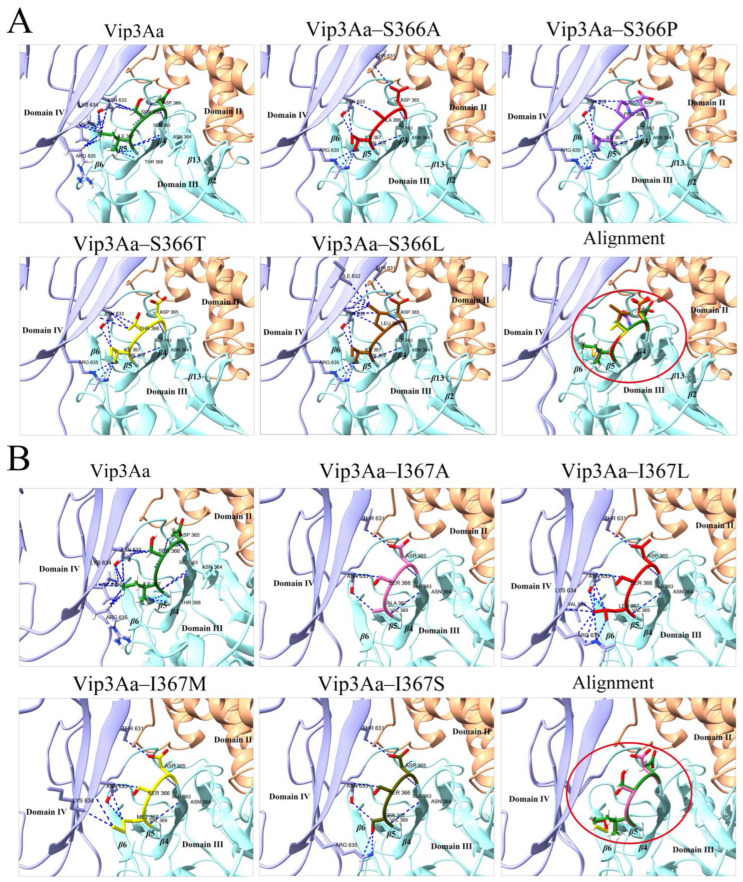
Contact analysis between residues by chimera X. (**A**) Vip3Aa and mutants of S366. (**B**) Vip3Aa and mutants of S367. Alignment indicates the result of comparison of *β*4–*β*5 loop in the structure of the Vip3Aa protein with that of the mutant proteins. The *β*4–*β*5 loop in Vip3Aa and its mutants were labeled with different colors. Blue: domain III; Purple: domain IV; Red circle: *β*4–*β*5 loop.

The contacts program in the software Chimera X v.1.8 was used to de–predict the contacts of the mutated residues with the surrounding residues in the 3D structures of the mutants. As shown in Figure 6A and Table S2, when serine at position 366 was replaced with alanine, proline, or threonine, 3D structural analysis of the mutants showed that the mutation not only reduced the contacts of the mutated residue with other residues but also significantly reduced the contacts of residues in the *β*4–*β*5 loop with other residues. Moreover, mutants at position 367 exhibited the same phenomenon (Figure 6B and Table S2). However, the 3D structure of Vip3Aa–S366L shows increased contacts between L366 and other residues (Figure 6A and Table S2). Additionally, we discovered that residue N633 in the 3D structure of the Vip3Aa protein forms nine contacts (four contacts with S366 and five contacts with I367) with residues in the *β*4–*β*5 loop (D^365^SI^367^) (Figure 6). Except for Vip3Aa–S366T and Vip3Aa–S366L, the contacts between residue N633 and residue at position 367 in the 3D structures of other mutants were no less than the contacts with residue at position 366.

**Figure 7:**
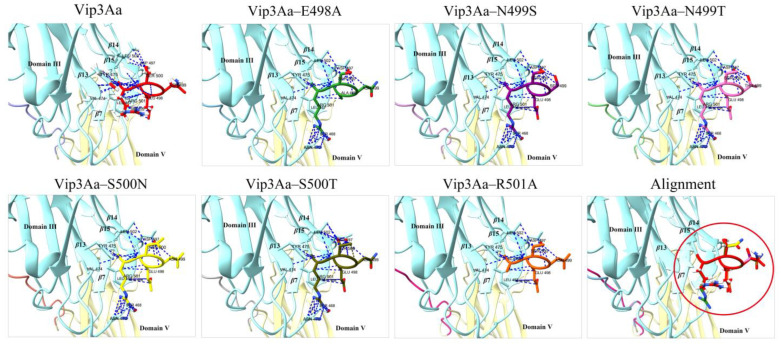
Contact analysis between residues in Vip3Aa (or mutants in the *β*14–*β*15 loop) by Chimera X. Alignment indicates the result of the comparison of the *β*14–*β*15 loop in the structure of the Vip3Aa protein with that of the mutant proteins. The *β*14–*β*15 loop in Vip3Aa and its mutants were labeled with different colors. Blue: domain III; Yellow: domain V; Red circle: *β*14–*β*15 loop.

Unlike the mutant Vip3Aa–R501A, it was not difficult to observe that the mutants with severely impaired toxicity (or insoluble mutant proteins) all contacted with N470, potentially restricting the flexibility of both the *β*14–*β*15 loop and *β*12–*β*13 loop (Figure 7 and Table S2). In addition, 3D structural analysis of the toxicity–enhanced mutant Vip3Aa–N470K suggests some contacts between K470 and R501 (Table S2). However, lysine and arginine are both positively charged amino acids and repel each other. This analysis shows that the *β*14–*β*15 loop and *β*12–*β*13 loop staying away from each other may assist in the toxicity of Vip3Aa.

## 3. Discussion

Vip3Aa, a soluble protein secreted by *B. thuringiensis*, can effectively control lepidopteran pests such as *S. frugiperda*. However, the lack of relevant research on the structure-function relationship of the Vip3Aa protein greatly hinders its application in pest control. The receptor binding function of domain III has been demonstrated but the specific regions involved in binding are still unclear. Therefore, we selected four specific loops (*β*4–*β*5 loop, *β*9–*β*10 loop, *β*12–*β*13 loop, and *β*14–*β*15 loop) in domain III as our research targets and constructed various mutants to investigate their insecticidal activity, proteolytic activity, and receptor binding ability. Additionally, we analyzed the impact of these loop mutations on the conformation of the Vip3Aa protein in an attempt to correlate structural features with insecticidal activity.

The correct folding is crucial for the Vip3Aa protein to exhibit insecticidal activity [8,21]. When the residues in the *β*9–*β*10 loop were all replaced with alanine, the mutant failed to form soluble protein, suggesting that the *β*9–*β*10 loop may be involved in the proper folding of Vip3Aa. To further analyze the effect of alanine substitution on Vip3Aa–loop9–10A, we predicted the 3D structure of Vip3Aa–loop9–10A using the Phyre2 server. The residues (K^429^KMKTL^434^) in the *β*9–*β*10 loop have 156 contacts with other residues but after replacing the residues in the *β*9–*β*10 loop with alanine, only 47 contacts are made (Figure S4). Additionally, there is a change in the size of the loop region before and after the mutation. In the structure of the Vip3Aa protein, most of the residues interacting with residues in the *β*9–*β*10 loop are located in domain III and some are located in domain V (Figure S4A).

The results of treating the mutants with trypsin or *S. frugiperda* midgut juice indicated that the stability of the other three mutants (Vip3Aa–loop4–5A, Vip3Aa–loop12–13A, and Vip3Aa–loop14–15A) was also affected but the impact on Vip3Aa–loop4–5A and Vip3Aa–loop14–15A was relatively minor compared to that on Vip3Aa–loop12–13A (Figure 2). This may be related to the larger regional span of the *β*12–*β*13 loop (Figure 1A). Even so, the percentage of 66 kDa fragments in Vip3Aa–loop12–13A was still higher than that of 75% of the activated fragments in Vip3Aa (Figure 2E,F). The affinity of Vip3Aa–loop4–5A, Vip3Aa–loop14–15A, or Vip3Aa–loop4–5A&14–15A for *S. frugiperda* BBMVs was significantly lower than that of Vip3Aa (Figure 3). Therefore, we speculate that the reason for the significant decrease in insecticidal activity of Vip3Aa–loop4–5A or Vip3Aa–loop14–15A is mainly related to the weakened affinity of the mutants for *S. frugiperda* BBMVs.

Currently, there is a lack of studies on the function of loops in the Vip3Aa protein. However, there are more reports on the roles of loops in Cry proteins. Loop 1 in domain II of the Cry2A toxin is involved in receptor recognition [22]. The Cry1Aa protein domain II loops contain binding sites for two functional receptors in the midgut of *Bombyx mori* and these binding sites overlap with each other [23]. Domain II loop 3 of Cry1Ab toxin is involved in a “ping pong” binding mechanism with *Manduca sexta* aminopeptidase–N and cadherin receptors [16]. Loops α–8 and 2 in domain II of Cry1Ab toxin interact with the Bt–R1 receptor in the midgut of *Manduca sexta* [24]. In this study, we found that Vip3Aa toxicity may be enhanced when N633 interacts more strongly with the residue at position 366 than with the residue at position 367 (*β*4–*β*5 loop: 364–DSI–368). However, N633 is located in domain IV, so the effect of these speculative residue interactions on Vip3Aa protein activity needs to be further investigated. In addition, ensuring the respective flexibility of both the *β*12–*β*13 loop and *β*14–*β*15 loop is indispensable in the insecticidal activity of Vip3Aa. However, we did not explore the binding of these loops to the identified Vip3Aa–interacting receptors (Sf–SR–C and Sf–FGFR) in Sf9 cells. Sf–SR–C and Sf–FGFR were identified as receptors for the action of Vip3Aa in Sf9 cells and were associated with cytotoxicity [19]. However, further investigation is needed to determine whether they are the receptors for Vip3Aa in the midgut of *S. frugiperda*. Our data (Figure 5) demonstrate that the affinity between the enhanced toxic mutants and *S. frugiperda* BBMVs is increased, indicating that the *β*4–*β*5 loop and *β*14–*β*15 loop in domain III are involved in the binding of Vip3Aa to its receptors. In addition, the affinity of Vip3Aa–loop 4–5A and Vip3Aa–loop 14–15A for *S. frugiperda* BBMVs, although decreased, was not lost and thus it is possible that other regions in domain III may be involved in the binding of Vip3Aa protein to the receptors. Mutants Vip3Aa–S366T and Vip3Aa–S366L showed significantly increased insecticidal toxicity compared to Vip3Aa. In the 3D structure of Vip3Aa–S366T or Vip3Aa–S366L, it was observed that N633 (located in domain IV) had more interactions with residue 366 rather than residue 367 in comparison to other *β*4–*β*5 mutants and Vip3Aa. Mutant Vip3Aa–N470K also shows enhanced insecticidal toxicity and the 3D structure of this mutant revealed a repulsion between residue K470 and residue R501. Furthermore, the *β*4–*β*5 loop, *β*12–*β*13 loop, and *β*14–*β*15 loop are all situated on one right side of domain III. Therefore, we propose that the loops on the right side of domain III play a role in the binding process of Vip3Aa to its receptors and that an appropriate increase in the distance between these prominent loops in domain III could facilitate the binding of Vip3Aa to its receptors.

After being ingested by insects, the Vip3Aa protein is activated by the intestinal juice and then binds to specific receptors, thereby exerting its insecticidal activity [7,10,25]. Therefore, increasing the activation efficiency of the Vip3Aa protein or enhancing its affinity for the receptors in the midgut may contribute to the insecticidal activity. Elucidation of the conformational relationship of the Vip3Aa protein is necessary to efficiently obtain Vip3Aa protein mutants with enhanced insecticidal activity. The increase in proteolytic cleavage sites between domain I and domain II resulted in the generation of a mutant (Vip3Aa^SS193RA/197RA^) with enhanced insecticidal activity against *S. frugiperda* [13]. Replacement of M34 in domain I with leucine increased the insecticidal activity against *S. exigua* and *S. littoralis* [12,26]. Mutants in domains IV and V showed improved structural stability and affinity for BBMVs and the insecticidal activity of these mutants against *S. frugiperda* and *Helicoverpa armigera* was also significantly improved [27]. In this study, we obtained single mutants that exhibited higher affinity for *S. frugiperda* BBMVs and showed increased insecticidal activity against *S. frugiperda* (Figure 2). However, except for Vip3Aa–S366T/N470K and Vip3Aa–S366L/N470K, the multiple mutants did not show improvement in insecticidal activity compared to the single mutants and even tended to decrease (Table 2 and Figure 4). Further investigation is needed to determine the specific reasons for this phenomenon. In addition, we also observed a curious phenomenon in our results, in which the replacement of individual residues with alanine in *β*4–*β*5 loop or *β*14–*β*15 loop largely attenuated the insecticidal toxicity of the Vip3Aa protein (S366A, I367A, and E498A), whereas the replacement of all the residues in *β*4–*β*5 loop or *β*14–*β*15 loop with alanine only reduced the toxicity to a certain extent (about 2–4 –fold). To explore the possible reasons for this phenomenon, we analyzed the contacts of residues in Vip3Aa and Vip3Aa mutants (Vip3Aa–loop 4–5A, Vip3Aa–S366A, Vip3Aa–I367A, Vip3Aa–loop 14–15A, and Vip3Aa–E498A) using Chimera X. As shown in Figure S5A, mutants Vip3Aa–S366A and Vip3Aa–I367A differ from Vip3Aa as well as Vip3Aa–loop 4–5A by increasing the contact of residue Asp365 with residue Thr631 (Figure S5B). However, as to whether these contacts are detrimental to the toxicity of Vip3Aa needs to be further investigated, since we can also observe the contacts of residue Asp365 with residue Thr631 in the mutant Vip3Aa–S366L.

## 4. Conclusions

In summary, we investigated the potential roles of four specific loops in domain III in the insecticidal activity of the Vip3Aa protein. The *β*9–*β*10 loop may be involved in the proper folding of Vip3Aa, while the other three loops (*β*4–*β*5 loop, *β*12–*β*13 loop, and *β*14–*β*15 loop) may be involved in the stability and insecticidal activity of Vip3Aa. Additionally, the *β*4–*β*5 loop and *β*14–*β*15 loop are involved in the receptor binding and insecticidal activity of Vip3Aa. Furthermore, maintaining the flexibility of the *β*14–*β*15 loop or appropriately increasing the spatial distance between the *β*14–*β*15 loop and *β*12–*β*13 loop also facilitates the enhancement of the Vip3Aa toxicity. Our findings are important for optimizing the insecticidal activity of Vip3Aa protein and shed light on the role of domain III in its functionality.

## 5. Materials and Methods

### 5.1. Plasmid Construction

The plasmid pET28a–Vip3Aa, which carries Vip3Aa11 (NCBI accession No. AAR36859), was used as the template to generate the expression plasmids for Vip3Aa mutants. The Vip3Aa protein structure (PDB: 6TFJ) reported by Núñez–Ramírez et al. was used as a reference for selecting amino acid mutation sites [7]. Two kinds of mutant vectors (loop replacement mutations and point mutations) were constructed in this study. Take the *β*4–*β*5 loop of Domain III in Vip3Aa as an example to expound the plasmid construction of loop replacement mutations. In brief, the loop sequence substitution (mutation of residues 364–DSI–368 to 364–AAA–368) was obtained by PCR amplification using the primers loop 4–5A–F/loop 4–5A–R. T4 ligase (TransGen Biotech, Beijing, China) was then used to ligate the phosphorylated PCR products to obtain the *β*4–*β*5 loop replacement mutant plasmid pET28a–Vip3Aa–loop 4–5A. All point mutation vectors were obtained using the Fast MultiSite Mutagenesis System (TransGen Biotech, Beijing, China). All primers used for plasmid construction in this study are listed in Table S1.

### 5.2. Protein Expression and Purification

Protein expression and purification were performed using the previously described method [28]. Briefly, *Escherichia coli* BL21 (DE3) cells harboring the mutant vectors were grown in LB broth medium supplemented with 50 μg kanamycin/mL at 37 °C with shaking until the OD600 reached 0.8–1.0. Then, the cell cultures were treated with 0.5 mM isopropyl–*β*–D–1–thiogalactopyranoside (IPTG) at 16 °C for 16–20 h. The target proteins were released from the cells by sonication and further purified with a Ni Sepharose^TM^ affinity column. The proteins were dialyzed in a buffer containing 25 mM Tris–HCl (pH 7.4) and 150 mM NaCl at 4 °C. The protein concentration was measured by the BCA Protein Quantitation Kit (Solarbio Science and Technology, Beijing, China).

### 5.3. Bioassay

*S. frugiperda* eggs and feed were purchased from Keyun Biological Pesticide (Zhengzhou, China). The toxicity of Vip3Aa mutant proteins to *S. frugiperda* neonate was tested by feeding with various concentrations (50, 100, 150, 300, 500, 1000, and 1500 ng/g) in a rearing chamber under a 16:8 h dark/light photocycle at 28 °C, with 50% relative humidity. The following are details about the major steps of the bioassay. First, the diet containing the toxin was divided into 24–well plates (~1 g per well) and the plates were placed at room temperature for approximately 2–3 h to dry the diet completely. Then, one *S. frugiperda* neonate larva was placed per well and the plates with larvae were cultured in the rearing chamber for seven days. Finally, the results of at least three independent trials were used to evaluate the mortality rate at each toxin dose. GraphPad Prism v.8.0 (GraphPad, San Diego, USA) was used to calculate the lethal concentration (LC_50_) values [27]. The toxicity of the proteins was quantified by probit analysis using SPSS Statistics (IBM, Chicago, IL, USA).

### 5.4. Proteolysis Assay

The preparation of midgut juice from *S. frugiperda* was carried out according to a protocol described elsewhere [29]. The protein concentration in midgut juice was determined by the BCA method. The trypsin used in this study was purchased from Solarbio Life Sciences (Beijing, China).

The purified protein (30 μg) and 10% trypsin were incubated at 37 °C for different durations (10 min, 20 min, 30 min, and 60 min). In addition, the purified proteins were treated with varying proportions (4%, 6%, 8%, and 10%) of the *S. frugiperda* midgut juice at 37 °C for 1 h. The protease inhibitor PMSF (1 mM) was used to terminate the hydrolysis reaction. Protein hydrolysis fragments were analyzed using SDS-PAGE.

### 5.5. ELISA Binding Assays

A saturation binding assay of protein to *S. frugiperda* BBMVs was performed as described by Yang et al. [27]. *S. frugiperda* BBMVs were prepared using the differential magnesium precipitation method [30]. The obtained BBMVs were rapidly frozen in liquid nitrogen and stored at −80 °C.

Briefly, a fixed amount of *S. frugiperda* BBMVs (1 μg) was added to each well of an ELISA plate (a 96–well plate) and incubated overnight at 4 °C. PBS buffer was used to remove the BBMVs that were not immobilized on the plate. After blocking with 2% bovine serum albumin (BSA), proteins labeled with biotin were added to the wells coated with BBMVs and incubated at 37 °C for 2 h. PBS buffer was used to remove the excess biotinylated protein. Streptavidin–horseradish peroxidase (1:20,000) was added to the wells and the plates were incubated at 37 °C for 1 h. Then, 3,3′,5,5′–tetramethylbenzidine substrate solution was added to each well and the plate was incubated for 10 min at 37 °C in the dark. Finally, the reaction was terminated by the addition of HCl (2 M) to each well. The results were measured at 450 nm using a microplate reader. The relative binding affinities were analyzed via Scatchard analysis with SigmaPlot v.14.0.

PBS buffer, BSA, streptavidin–horseradish peroxidase, and 3,3′,5,5′–tetramethylbenzidine were purchased from Solarbio Life Sciences (Beijing, China).

### 5.6. Vip3Aa or Mutant Subcellular Localization in Sf9 Cells

The subcellular localization of Vip3Aa or mutants in Sf9 cells was described in our previous study [28]. Briefly, Vip3Aa (or mutant proteins) with red fluorescent protein (RFP) was used to treat Sf9 cells for 6 h. After washing with PBS buffer three times, the Sf9 cells were stained with Dio dye (Beyotime, Shanghai, China) at 28 °C in the dark for 45 min. Then, the cells were imaged with a Zeiss LSM710 fluorescence microscope.

### 5.7. Protein Structure Modeling and Analysis

The Phyre2 server was used to predict the three–dimensional (3D) structure of the mutants [31]. Chimera X was used to analyze the contacts of residues [32].

### 5.8. Statistical Analysis

The significance was tested using one–way analysis of variance using Student’s t–test. If the *p* value was ≤0.05, the results were considered significant. All experiments were performed with at least three biological replicates and technical replicates.

## Figures and Tables

**Table 1:** Toxicity of the Vip3Aa and loop mutant proteins against *S. frugiperda* neonate larvae.

Protein	Mutation Description	LC_50_ (ng/g) (95% Fiducial Limits)	Slope ± SE	χ^2^	DF
Vip3Aa	Wild type, no mutation	251 (226–307)	2.42 ± 0.15	8.97	5
Vip3Aa–loop 4–5A	Mutation of residues 364–DSI–368 to 364–AAA–368	594 (506–713)	1.56 ± 0.12	0.38	5
Vip3Aa–loop12–13A	Mutation of residues 467–SANDDG–474 to 467–AAAAAA–474	375 (330–427)	2.00 ± 0.13	1.55	5
Vip3Aa–loop14–15A	Mutation of residues 497–ENSR–502 to 497–AAAA–502	970 (805–1219)	1.59 ± 0.13	1.58	5

**Table 2:** Toxicity of modified Vip3Aa proteins against *S. frugiperda* neonate larvae.

Protein	Position	LC_50_ (ng/g) (95% Fiducial Limits)
Vip3Aa	-	251 (226–307)
Vip3Aa–N470K	*β*12–*β*13 loop	123 (107–141)
Vip3Aa–S366T/R501A	*β*4–*β*5 loop and *β*14–*β*15 loop	145 (122–168)
Vip3Aa–S366L/R501A		128 (104–172)
Vip3Aa–S366T/N470K	*β*4–*β*5 loop and *β*12–*β*13 loop	68 (51–86)
Vip3Aa–S366L/N470K		56 (39–77)
Vip3Aa–N470K/R501A	*β*12–*β*13 loop and *β*14–*β*15 loop	113 (106–131)
Vip3Aa–S366T/N470K/R501A	*β*4–*β*5 loop, *β*12–*β*13 loop and *β*14–*β*15 loop	108 (92–122)
Vip3Aa–S366L/N470K/R501A		106 (86–125)

## Data Availability

All original research data supporting reported results can be made available upon request.

## Supplementary Materials

The following supporting information can be downloaded at https://www.mdpi.com/article/10.3390/toxins16010023/s1. Table S1: Primers used in this study; Table S2: Analysis of residue interactions; Table S3. Contact residues analysis with residues in *β*9–*β*10 loop; Figure S1: Alignment of the amino acid sequences in domain III; Figure S2: The four special loops exposed on the surface of the Vip3Aa protein domain III; Figure S3: Western blot analysis of the binding between Vip3Aa or its mutants and *S. frugiperda* BBMVs; Figure S4: Interaction analysis between residues by chimera X. Figure S5: Contacts analysis between residues by chimera X. (A) Vip3Aa and *β*4–*β*5 loop mutants. (B) Vip3Aa and *β*14–*β*15 loop mutants. Green: domain III, pink: domain IV.
