# Supplementary material for: Domain III β4–β5 Loop and β14–β15 Loop of Bacillus thuringiensis Vip3Aa Are Involved in Receptor Binding and Toxicity
Source: Toxins (Basel). 2024 Jan 1;16(1):23. doi: 10.3390/toxins16010023 (PMC10820090; doi:10.3390/toxins16010023)
Supplement: Supplementary file 1 [file toxins-16-00023-s001.zip › toxins-2751484-supplementary.pdf]

Supplementary Materials

Domain III β4–β5 Loop and β14–β15 Loop of *Bacillus thuringiensis* Vip3Aa Are Involved in Receptor Binding and Toxicity

Xiaoyue Hou [1,2,3,4,5], Mengjiao Li [4], Chengjuan Mao [4], Lei Jiang [4], Wen Zhang [4], Mengying Li [4], Xiaomeng Geng [4], Xin Li [4], Shu Liu [1,2,3,4], Guang Yang [1,2,3,4], Jing Zhou [6], Yaowei Fang [1,2,3,4,*] and Jun Cai [5,*]

[1] Jiangsu Key Laboratory of Marine Bioresources and Environment, Jiangsu Ocean University, Lianyungang 222005, China; 2020000057@jou.edu.cn (X.H.); 2007000028@jou.edu.cn (S.L.); 2020000050@jou.edu.cn (G.Y.)

[2] Co–Innovation Center of Jiangsu Marine Bio–Industry Technology, Jiangsu Ocean University, Lianyungang 222005, China

[3] Jiangsu Marine Resources Development Research Institute, Jiangsu Ocean University, Lianyungang 222005, China

[4] College of Marine Food and Bioengineering, Jiangsu Ocean University, Lianyungang 222005, China; xydxhl@163.com (M.L.); mcj15055202109@163.com (C.M.); jl17892521856@163.com (L.J.); zw19975066385@163.com (W.Z.); lmy13775446376@163.com (M.L.); gxm18913965276@163.com (X.G.); 2021122077@jou.edu.cn (X.L.)

[5] College of Life Sciences, Nankai University, Tianjin 300071, China

[6] Lianyungang City Quality Technology Comprehensive Inspection and Quality Inspection Center, Lianyungang 222346, China; zj13179573851@163.com

* Correspondence: 2007000027@jou.edu.cn (Y.F.); caijun@nankai.edu.cn (J.C.)

Table S1. Primers used in this study.

Primers	Primer Sequence
Loop4/5A–F	GCTGCAACAGTATTAAAAGTATATGAGGCT
Loop4/5A–R	GGCATTACTAATTTCAAACCCAATCAAT
Loop9/10A–F	GCTGCAGCTAGATATGAGGTAACAGCGAATTTT
Loop9/10A–R	GGCTGCGGCAGTGAAATCAATTTTAGTAATTAC
Loop12/13A–F	GCTGCAGCTGTGTATATGCCGTTAGGTGTC
Loop12/13A–R	GGCTGCGGCTAACGTTCTATACTCCGCTTC
Loop14/15A–F	GCCGCATTAATTACTTTAACATGTAAATC
Loop14/15A–R	GGCTGCATCAGCTTGGAGGCCAAAC
D365A–F	TTTGAAATTAGTAATGCTTCAATTACAGTAT
D365A–R	GCATTACTAATTTCAAACCCAATCAATGCAT
S366A–F	TGAAATTAGTAATGATGCAATTACAGTATTAAAAG
S366A–R	CATCATTACTAATTTCAAACCCAATCAATGCATG
I367A–F	GAAATTAGTAATGATTCAGCTACAGTATTAAAAGT
I367A–R	CGTGAATCATTACTAATTTCAAACCCAATCAATGC
D365N–F	TTTGAAATTAGTAATAATTCAATTACAGTAT
D365N–R	TATTACTAATTTCAAACCCAATCAATGCAT
S366P–F	TGAAATTAGTAATGATCCAATTACAGTATTAAAAG
S366P–R	GATCATTACTAATTTCAAACCCAATCAATGCATG
S366R–F	GGTTTGAAATTAGTAATGATAGAATTACAGTATT
S366R–R	CTATCATTACTAATTTCAAACCCAATCAATGCAT
S366D–F	TGAAATTAGTAATGATGACATTACAGTATTAAAAG
S366D–R	GTCATCATTACTAATTTCAAACCCAATCAATGCAT
S366E–F	TGAAATTAGTAATGATGAAATTACAGTATTAAAAG
S366E–R	TCATCATTACTAATTTCAAACCCAATCAATGCATG
S366T–F	GAAATTAGTAATGATACAATTACAGTATTAAAAG
S366T–R	TATCATTACTAATTTCAAACCCAATCAATGCATG
S366N–F	GAAATTAGTAATGATAACATTACAGTATTAAAAG
S366N–R	GTTATCATTACTAATTTCAAACCCAATCAATGCAT
S366L–F	GAAATTAGTAATGATTTAATTACAGTATTAAAAG
S366L–R	AAATCATTACTAATTTCAAACCCAATCAATGCAT
S366K–F	GGTTTGAAATTAGTAATGATAAAATTACAGTATT
S366K–R	TTATCATTACTAATTTCAAACCCAATCAATGCAT
I367S–F	GAAATTAGTAATGATTCAAGTACAGTATTAAAAGT
I367S–R	CTTGAATCATTACTAATTTCAAACCCAATCAATGC
I367M–F	GAAATTAGTAATGATTCAAGTACAGTATTAAAAGT
I367M–R	CATTGAATCATTACTAATTTCAAACCCAATCAATGC
I367L–F	GAAATTAGTAATGATTCAGTTACAGTATTAAAAG

I367L–R	CTGAATCATTACTAATTTCAAACCCAATCAATGC
N470K–F	GAACGTTAAGTGCTAAGGATGATGGGGTGTAT
N470K–R	CTTAGCACTTAACGTTCTATACTCCGCTTCAC
E498A–F	GGCCTCCAAGCTGATGCAAATTCAAGATTAATTAC
E498A–R	GCATCAGCTTGGAGGCCAAACCCATTAATC
N499A–F	CCTCCAAGCTGATGAAGCTTCAAGATTAATTAC
N499A–R	GCTTCATCAGCTTGGAGGCCAAACCCATTAAT
S500A–F	CCAAGCTGATGAAAATGCAAGATTAATTACTTT
S500A–R	CATTTTCATCAGCTTGGAGGCCAAACCCATTAAT
R501A–F	CAAGCTGATGAAAATTCAGCATTAATTACTTTAAC
R501A–R	GCTGAATTTTCATCAGCTTGGAGGCCAAACCCATTAAT
E498K–F	TTGGCCTCCAAGCTGATAAAAATTCAAGATTAATT
E498K–R	TATCAGCTTGGAGGCCAAACCCATTAATCGGAGTC
N499K–F	CCAAGCTGATGAAAAGTCAAGATTAATTACTTT
N499K–R	CTTTTCATCAGCTTGGAGGCCAAACCCATTAATC
N499R–F	CTCCAAGCTGATGAACGTTCAAGATTAATTACT
N499R–R	CGTTCATCAGCTTGGAGGCCAAACCCATTAATC
N499T–F	CTCCAAGCTGATGAAACTTCAAGATTAATTAC
N499T–R	GTTTCATCAGCTTGGAGGCCAAACCCATTAAT
N499D–F	CTCCAAGCTGATGAAGATTCAAGATTAATTAC
N499D–R	CTTCATCAGCTTGGAGGCCAAACCCATTAAT
N499S–F	CTCCAAGCTGATGAAAGTTCAAGATTAATTAC
N499S–R	CTTTCATCAGCTTGGAGGCCAAACCCATTAAT
S500T–F	CAAGCTGATGAAAATACAAGATTAATTACTT
S500T–R	TATTTTCATCAGCTTGGAGGCCAAACCCATT
S500N–F	CCAAGCTGATGAAAATAATAGATTAATTACTTT
S500N–R	ATTATTTTCATCAGCTTGGAGGCCAAACCCATT
R501K–F	CTGATGAAAATTCAAAATTAATTACTTTAAC
R501K–R	TTTGAATTTTCATCAGCTTGGAGGCCAAACC
R501Q–F	CAAGCTGATGAAAATTCACAATTAATTACTTTAAC
R501Q–R	TGTGAATTTTCATCAGCTTGGAGGCCAAACCCATTAAT

Table S2. Contact residues analysis with residues in $\beta 4$–$\beta 5$ loop、$\beta 14$–$\beta 15$ loop and $\beta 12$–$\beta 13$ loop.

Protein	Loop/residues	Contacts	Atom1	Atom2	Overlap	Distance
Vip3Aa	$\beta 4$–$\beta 5$ loop ($D^{365}SI^{367}$)	24	ILE 367 CD1	ASN 633 OD1	0.184	2.996
			ILE 367 O	SER 363 O	0.081	2.879
			ILE 367 HG22	THR 368 N	0.079	2.546
			ILE 367 HD13	ARG 635 CG	0.031	2.669
			ILE 367 HD12	ASN 633 OD1	0.03	2.45
			SER 366 CB	ASN 633 HB3	–0.052	2.752
			ILE 367 CD1	ARG 635 CG	–0.103	3.503
			SER 366 HB2	ASN 633 HB3	–0.128	2.128
			SER 366 HB2	ASN 633 CB	–0.133	2.833
			SER 366 CB	ASN 633 CB	–0.154	3.554
			ILE 367 CG2	THR 368 H	–0.167	2.867
			ILE 367 CD1	ASN 633 CG	–0.169	3.569
			ILE 367 HD13	ARG 635 HG3	–0.242	2.242
			ILE 367 HD11	ASN 633 OD1	–0.269	2.749
			ILE 367 O	ASN 364 HA	–0.271	2.751
			ILE 367 CD1	ARG 635 HG3	–0.272	2.972
			ASP 365 O	ASN 364 O	–0.315	3.275
			SER 366 H	ILE 367 H	–0.32	2.32
			ILE 367 HD12	VAL 596 CG1	–0.33	3.03
			ASP 365 HB2	SER 366 H	–0.344	2.344
			ILE 367 HD11	LYS 634 C	–0.357	3.057

		ILE 367 CD1	LYS 634 C	–0.375	3.775
		ILE 367 HD12	ASN 633 CG	–0.384	3.084
		ILE 367 HD13	ARG 635 HG2	–0.385	2.385
S366	6	SER 366 CB	ASN 633 HB3	–0.052	2.752
		SER 366 HB2	ASN 633 HB3	–0.128	2.128
		SER 366 HB2	ASN 633 CB	–0.133	2.833
		SER 366 CB	ASN 633 CB	–0.154	3.554
		SER 366 H	ILE 367 H	–0.32	2.32
		SER 366 H	ASP 365 HB2	–0.344	2.344
I367	18	ILE 367 CD1	ASN 633 OD1	0.184	2.996
		ILE 367 O	SER 363 O	0.081	2.879
		ILE 367 HG22	THR 368 N	0.079	2.546
		ILE 367 HD13	ARG 635 CG	0.031	2.669
		ILE 367 HD12	ASN 633 OD1	0.03	2.45
		ILE 367 CD1	ARG 635 CG	–0.103	3.503
		ILE 367 CG2	THR 368 H	–0.167	2.867
		ILE 367 CD1	ASN 633 CG	–0.169	3.569
		ILE 367 HD13	ARG 635 HG3	–0.242	2.242
		ILE 367 HD11	ASN 633 OD1	–0.269	2.749
		ILE 367 O	ASN 364 HA	–0.271	2.751
		ILE 367 CD1	ARG 635 HG3	–0.272	2.972
		ILE 367 H	SER 366 H	–0.32	2.32
		ILE 367 HD12	VAL 596 CG1	–0.33	3.03

			ILE 367 HD11	LYS 634 C	–0.357	3.057
			ILE 367 CD1	LYS 634 C	–0.375	3.775
			ILE 367 HD12	ASN 633 CG	–0.384	3.084
			ILE 367 HD13	ARG 635 HG2	–0.385	2.385
Vip3Aa–S366A	β4–β5 loop ($D^{365}AI^{367}$)	11	ILE 367 CG2	ARG 635 NH1	0.865	2.655
			ILE 367 CG2	ARG 635 NE	0.445	3.075
			ILE 367 CG2	ARG 635 CZ	0.225	3.265
			ILE 367 CG1	ASN 633 OD1	0.212	3.088
			ALA 366 CB	ASN 633 CB	0.144	3.616
			ILE 367 CG2	VAL 369 CG1	0.072	3.688
			ILE 367 O	SER 363 O	–0.087	2.927
			ASP 365 OD2	THR 631 CG2	–0.225	3.525
			ILE 367 CG1	ASN 633 CG	–0.229	3.719
			ILE 367 CB	VAL 369 CG1	–0.262	4.022
			ASP 365 O	ASN 364 O	–0.339	3.179
	A366	1	ALA 366 CB	ASN 633 CB	0.144	3.616
Vip3Aa–S366P	β4–β5 loop ($D^{365}PI^{367}$)	13	ILE 367 CG2	ARG 635 NH1	0.865	2.655
			ILE 367 CG2	ARG 635 NE	0.445	3.075
			PRO 366 CB	ASN 633 CB	0.229	3.531
			ILE 367 CG2	ARG 635 CZ	0.225	3.265
			ILE 367 CG1	ASN 633 OD1	0.212	3.088
			PRO 366 CG	ASN 633 CB	0.155	3.605
			ILE 367 CG2	VAL 369 CG1	0.072	3.688

			ASP 365 CB	PRO 366 CG	0.006	3.754
			ASP 365 CG	PRO 366 CD	–0.086	3.846
			ILE 367 O	SER 363 O	–0.087	2.927
			ILE 367 CG1	ASN 633 CG	–0.229	3.719
			ILE 367 CB	VAL 369 CG1	–0.262	4.022
			ASP 365 O	ASN 364 O	–0.339	3.179
	P366	4	PRO 366 CB	ASN 633 CB	0.229	3.531
			PRO 366 CG	ASN 633 CB	0.155	3.605
			PRO 366 CG	ASP 365 CB	0.006	3.754
			PRO 366 CD	ASP 365 CG	–0.086	3.846
Vip3Aa–S366T	*β*4–*β*5 loop ($D^{365}TI^{367}$)	12	ILE 367 CG2	ARG 635 NH1	0.865	2.655
			ILE 367 CG2	ARG 635 NE	0.445	3.075
			ILE 367 CG2	ARG 635 CZ	0.225	3.265
			ILE 367 CG1	ASN 633 OD1	0.212	3.088
			THR 366 CB	ASN 633 CB	0.151	3.609
			ILE 367 CG2	VAL 369 CG1	0.072	3.688
			ILE 367 O	SER 363 O	–0.087	2.927
			ILE 367 CG1	ASN 633 CG	–0.229	3.719
			THR 366 CG2	ASN 633 CB	–0.247	4.007
			ILE 367 CB	VAL 369 CG1	–0.262	4.022
			THR 366 CG2	ASN 633 O	–0.275	3.575
			ASP 365 O	ASN 364 O	–0.339	3.179
	T366	3	THR 366 CB	ASN 633 CB	0.151	3.609
			THR 366 CG2	ASN 633 CB	–0.247	4.007

			THR 366 CG2	ASN 633 O	–0.275	3.575
Vip3Aa–S366L	*β*4–*β*5 loop (D^{365}LI^{367})	20	ILE 367 CG2	ARG 635 NH1	0.865	2.655
			LEU 366 CD1	ASN 633 N	0.612	2.908
			LEU 366 CD1	ASN 633 CB	0.544	3.216
			LEU 366 CD1	THR 631 O	0.474	2.826
			ILE 367 CG2	ARG 635 NE	0.445	3.075
			ILE 367 CG2	ARG 635 CZ	0.225	3.265
			ILE 367 CG1	ASN 633 OD1	0.212	3.088
			LEU 366 CD1	ASN 633 CA	0.167	3.593
			LEU 366 CB	ASN 633 CB	0.15	3.61
			ILE 367 CG2	VAL 369 CG1	0.072	3.688
			LEU 366 CD1	ILE 632 CA	–0.006	3.766
			ILE 367 O	SER 363 O	–0.087	2.927
			LEU 366 CD1	ILE 632 C	–0.094	3.584
			LEU 366 CG	ASN 633 CB	–0.166	3.926
			ASP 365 OD2	THR 631 CG2	–0.225	3.525
			ILE 367 CG1	ASN 633 CG	–0.229	3.719
			ILE 367 CB	VAL 369 CG1	–0.262	4.022
			LEU 366 CD1	THR 631 C	–0.266	3.756
			ASP 365 O	ASN 364 O	–0.339	3.179
			ASP 365 O	LEU 366 CD2	–0.383	3.683
	L366	10	LEU 366 CD1	ASN 633 N	0.612	2.908
			LEU 366 CD1	ASN 633 CB	0.544	3.216
			LEU 366 CD1	THR 631 O	0.474	2.826

			LEU 366 CD1	ASN 633 CA	0.167	3.593
			LEU 366 CB	ASN 633 CB	0.15	3.61
			LEU 366 CD1	ILE 632 CA	–0.006	3.766
			LEU 366 CD1	ILE 632 C	–0.094	3.584
			LEU 366 CG	ASN 633 CB	–0.166	3.926
			LEU 366 CD1	THR 631 C	–0.266	3.756
			LEU 366 CD2	ASP 365 O	–0.383	3.683
Vip3Aa–I367A	β4–β5 loop ($D^{365}SA^{367}$)	6	SER 366 CB	ASN 633 CB	0.15	3.61
			ALA 367 O	SER 363 O	–0.087	2.927
			ALA 367 CB	ASN 633 OD1	–0.16	3.46
			ASP 365 OD2	THR 631 CG2	–0.225	3.525
			ALA 367 CB	VAL 369 CG1	–0.268	4.028
			ASP 365 O	ASN 364 O	–0.339	3.179
	A367	3	ALA 367 O	SER 363 O	–0.087	2.927
			ALA 367 CB	ASN 633 OD1	–0.16	3.46
			ALA 367 CB	VAL 369 CG1	–0.268	4.028
Vip3Aa–I367L	β4–β5 loop ($D^{365}SL^{367}$)	16	LEU 367 CD1	ASN 633 OD1	0.771	2.529
			LEU 367 CD1	ARG 635 CG	0.489	3.271
			LEU 367 CD1	VAL 596 CG1	0.344	3.416
			LEU 367 CG	ASN 633 OD1	0.266	3.034
			SER 366 CB	ASN 633 CB	0.15	3.61
			LEU 367 CD1	ARG 635 NE	–0.017	3.537
			LEU 367 CD1	ASN 633 CG	–0.065	3.555
			LEU 367 O	SER 363 O	–0.087	2.927

			LEU 367 CG	ASN 633 CG	–0.207	3.697
			LEU 367 CD1	ARG 635 CD	–0.213	3.973
			ASP 365 OD2	THR 631 CG2	–0.225	3.525
			LEU 367 CB	VAL 369 CG1	–0.264	4.024
			LEU 367 CD2	ARG 635 NE	–0.303	3.823
			LEU 367 CD1	LYS 634 C	–0.314	3.804
			ASP 365 O	ASN 364 O	–0.339	3.179
			LEU 367 CD1	LYS 634 O	–0.352	3.652
	L367	13	LEU 367 CD1	ASN 633 OD1	0.771	2.529
			LEU 367 CD1	ARG 635 CG	0.489	3.271
			LEU 367 CD1	VAL 596 CG1	0.344	3.416
			LEU 367 CG	ASN 633 OD1	0.266	3.034
			LEU 367 CD1	ARG 635 NE	–0.017	3.537
			LEU 367 CD1	ASN 633 CG	–0.065	3.555
			LEU 367 O	SER 363 O	–0.087	2.927
			LEU 367 CG	ASN 633 CG	–0.207	3.697
			LEU 367 CD1	ARG 635 CD	–0.213	3.973
			LEU 367 CB	VAL 369 CG1	–0.264	4.024
			LEU 367 CD2	ARG 635 NE	–0.303	3.823
			LEU 367 CD1	LYS 634 C	–0.314	3.804
			LEU 367 CD1	LYS 634 O	–0.352	3.652
Vip3Aa–I367S	β4–β5 loop ($D^{365}SS^{367}$)	9	SER 366 CB	ASN 633 CB	0.15	3.61
			SER 367 O	SER 363 O	–0.087	2.927
			SER 367 OG	ARG 635 NH1	–0.109	2.809

			SER 367 CB	ASN 633 OD1	–0.158	3.458
			ASP 365 OD2	THR 631 CG2	–0.225	3.525
			SER 367 OG	VAL 369 CG1	–0.26	3.6
			SER 367 CB	VAL 369 CG1	–0.264	4.024
			ASP 365 O	ASN 364 O	–0.339	3.179
			SER 367 OG	ARG 635 CZ	–0.343	3.413
	S367	6	SER 367 O	SER 363 O	–0.087	2.927
			SER 367 OG	ARG 635 NH1	–0.109	2.809
			SER 367 CB	ASN 633 OD1	–0.158	3.458
			SER 367 OG	VAL 369 CG1	–0.26	3.6
			SER 367 CB	VAL 369 CG1	–0.264	4.024
			SER 367 OG	ARG 635 CZ	–0.343	3.413
Vip3Aa–I367M	β4–β5 loop ($D^{365}SM^{367}$)	9	MET 367 CG	ASN 633 OD1	0.203	3.097
			SER 366 CB	ASN 633 CB	0.15	3.61
			MET 367 O	SER 363 O	–0.087	2.927
			MET 367 CE	ASN 633 O	–0.2	3.5
			MET 367 CE	LYS 634 CA	–0.222	3.982
			ASP 365 OD2	THR 631 CG2	–0.225	3.525
			MET 367 CG	ASN 633 CG	–0.262	3.752
			MET 367 CB	VAL 369 CG1	–0.264	4.024
			ASP 365 O	ASN 364 O	–0.339	3.179
	M367	6	MET 367 CG	ASN 633 OD1	0.203	3.097
			MET 367 O	SER 363 O	–0.087	2.927
			MET 367 CE	ASN 633 O	–0.2	3.5

			MET 367 CE	LYS 634 CA	–0.222	3.982
			MET 367 CG	ASN 633 CG	–0.262	3.752
			MET 367 CB	VAL 369 CG1	–0.264	4.024
Vip3Aa	β14–β15 loop ($E^{498}NSR^{501}$)	36	ARG 501 CA	ASP 497 O	0.353	2.827
			GLU 498 CD	THR 466 O	0.318	2.862
			ARG 501 N	ASP 497 O	0.149	2.556
			GLU 498 HA	LEU 467 CD2	0.085	2.615
			ARG 501 HA	ASP 497 O	0.075	2.405
			ARG 501 H	ASP 497 O	0.015	2.065
			ARG 501 O	VAL 474 HG13	–0.002	2.482
			SER 500 O	ARG 501 HB2	–0.01	2.49
			GLU 498 OE1	THR 466 O	–0.042	3.002
			ARG 501 O	TYR 475 HB2	–0.051	2.531
			SER 500 OG	LEU 502 CG	–0.079	3.279
			SER 500 OG	LEU 502 CD1	–0.087	3.287
			GLU 498 CA	LEU 467 CD2	–0.094	3.494
			SER 500 OG	LEU 502 HG	–0.104	2.604
			ARG 501 O	VAL 474 CG1	–0.123	3.303
			ARG 501 O	TYR 475 HD2	–0.126	2.606
			ARG 501 C	ASP 497 O	–0.149	3.329
			ARG 501 O	TYR 475 H	–0.156	2.236
			GLU 498 OE2	THR 466 O	–0.164	3.124
			ARG 501 O	TYR 475 CB	–0.171	3.351
			ARG 501 H	SER 500 H	–0.181	2.181

		GLU 498 CG	THR 466 O	–0.195	3.375
		SER 500 OG	LEU 502 HD12	–0.225	2.725
		GLU 498 C	ARG 501 H	–0.229	2.929
		GLU 498 C	SER 500 H	–0.25	2.95
		ARG 501 O	TYR 475 CD2	–0.262	3.442
		SER 500 O	TYR 475 CD2	–0.293	3.473
		ARG 501 H	ASP 497 C	–0.308	3.008
		ARG 501 N	ASP 497 C	–0.317	3.642
		SER 500 O	TYR 475 HD2	–0.34	2.82
		GLU 498 HA	LEU 467 HD23	–0.347	2.347
		ARG 501 HA	LEU 467 CD2	–0.357	3.057
		ARG 501 O	TYR 475 N	–0.358	3.063
		GLU 498 CA	LEU 467 HD23	–0.374	3.074
		SER 500 C	LEU 502 H	–0.385	3.085
		SER 500 O	ARG 501 O	–0.399	3.359
E498	10	GLU 498 CD	THR 466 O	0.318	2.862
		GLU 498 HA	LEU 467 CD2	0.085	2.615
		GLU 498 OE1	THR 466 O	–0.042	3.002
		GLU 498 CA	LEU 467 CD2	–0.094	3.494
		GLU 498 OE2	THR 466 O	–0.164	3.124
		GLU 498 CG	THR 466 O	–0.195	3.375
		GLU 498 C	ARG 501 H	–0.229	2.929
		GLU 498 C	SER 500 H	–0.25	2.95
		GLU 498 HA	LEU 467 HD23	–0.347	2.347

		GLU 498 CA	LEU 467 HD23	–0.374	3.074
N499	0	/	/	/	/
S500	11	SER 500 O	ARG 501 HB2	–0.01	2.49
		SER 500 OG	LEU 502 CG	–0.079	3.279
		SER 500 OG	LEU 502 CD1	–0.087	3.287
		SER 500 OG	LEU 502 HG	–0.104	2.604
		SER 500 H	ARG 501 H	–0.181	2.181
		SER 500 OG	LEU 502 HD12	–0.225	2.725
		SER 500 H	GLU 498 C	–0.25	2.95
		SER 500 O	TYR 475 CD2	–0.293	3.473
		SER 500 O	TYR 475 HD2	–0.34	2.82
		SER 500 C	LEU 502 H	–0.385	3.085
		SER 500 O	ARG 501 O	–0.399	3.359
R501	20	ARG 501 CA	ASP 497 O	0.353	2.827
		ARG 501 N	ASP 497 O	0.149	2.556
		ARG 501 HA	ASP 497 O	0.075	2.405
		ARG 501 H	ASP 497 O	0.015	2.065
		ARG 501 O	VAL 474 HG13	–0.002	2.482
		ARG 501 HB2	SER 500 O	–0.01	2.49
		ARG 501 O	TYR 475 HB2	–0.051	2.531
		ARG 501 O	VAL 474 CG1	–0.123	3.303
		ARG 501 O	TYR 475 HD2	–0.126	2.606
		ARG 501 C	ASP 497 O	–0.149	3.329
		ARG 501 O	TYR 475 H	–0.156	2.236

			ARG 501 O	TYR 475 CB	–0.171	3.351
			ARG 501 H	SER 500 H	–0.181	2.181
			ARG 501 H	GLU 498 C	–0.229	2.929
			ARG 501 O	TYR 475 CD2	–0.262	3.442
			ARG 501 H	ASP 497 C	–0.308	3.008
			ARG 501 N	ASP 497 C	–0.317	3.642
			ARG 501 HA	LEU 467 CD2	–0.357	3.057
			ARG 501 O	TYR 475 N	–0.358	3.063
			ARG 501 O	SER 500 O	–0.399	3.359
Vip3Aa–E498A	*β*14–*β*15 loop ($A^{498}NSR^{501}$)	31	ARG 501 CA	ASP 497 O	0.464	2.836
			ARG 501 NH1	ASN 470 CB	0.287	3.233
			ARG 501 NH2	SER 468 O	0.233	2.427
			ARG 501 CZ	ASN 470 CB	0.2	3.29
			ALA 498 CA	LEU 467 CD2	0.16	3.6
			ARG 501 NH1	SER 468 OG	0.132	2.568
			ARG 501 N	ASP 497 O	0.117	2.543
			SER 500 CB	LEU 502 CG	0.098	3.662
			ARG 501 O	VAL 474 CG1	0.044	3.256
			ARG 501 NE	ASN 470 CB	–0.102	3.622
			SER 500 CB	LEU 502 CD1	–0.127	3.887
			ARG 501 O	TYR 475 CB	–0.165	3.465
			ASN 499 CB	ASP 497 OD1	–0.182	3.482
			ARG 501 NH2	SER 468 C	–0.191	3.441
			ASN 499 N	ASP 497 OD1	–0.204	2.864

			ARG 501 CA	LEU 467 CD2	–0.207	3.967
			ARG 501 NH2	ASN 470 CB	–0.245	3.765
			ARG 501 NH1	ASN 470 ND2	–0.249	3.529
			ALA 498 N	ASP 497 OD1	–0.251	2.911
			ARG 501 CZ	SER 468 O	–0.252	3.282
			ARG 501 NH2	SER 468 N	–0.277	3.557
			ASN 499 CA	ASP 497 OD1	–0.293	3.593
			ARG 501 C	ASP 497 O	–0.305	3.335
			ARG 501 O	TYR 475 CD1	–0.314	3.494
			ALA 498 CB	LEU 467 CD2	–0.317	4.077
			SER 500 N	ASP 497 CG	–0.353	3.873
			ARG 501 NH1	SER 468 CB	–0.354	3.874
			ARG 501 CZ	SER 468 OG	–0.372	3.442
			ARG 501 N	ASP 497 C	–0.396	3.646
			SER 500 O	LEU 502 CG	–0.396	3.696
			SER 500 O	TYR 475 CD1	–0.397	3.577
	A498	3	ALA 498 CA	LEU 467 CD2	0.16	3.6
			ALA 498 N	ASP 497 OD1	–0.251	2.911
			ALA 498 CB	LEU 467 CD2	–0.317	4.077
Vip3Aa–N499T	β14–β15 loop ($E^{498}TSR^{501}$)	36	ARG 501 CA	ASP 497 O	0.464	2.836
			GLU 498 CD	THR 466 O	0.392	2.908
			ARG 501 NH1	ASN 470 CB	0.287	3.233
			ARG 501 NH2	SER 468 O	0.233	2.427
			ARG 501 CZ	ASN 470 CB	0.2	3.29

	GLU 498 CA	LEU 467 CD2	0.16	3.6
	ARG 501 NH1	SER 468 OG	0.132	2.568
	ARG 501 N	ASP 497 O	0.117	2.543
	THR 499 OG1	ASP 497 CG	0.105	3.235
	SER 500 CB	LEU 502 CG	0.098	3.662
	GLU 498 OE1	THR 466 O	0.089	2.751
	ARG 501 O	VAL 474 CG1	0.044	3.256
	GLU 498 CG	THR 466 O	–0.056	3.356
	THR 499 OG1	ASP 497 OD1	–0.068	2.548
	ARG 501 NE	ASN 470 CB	–0.102	3.622
	SER 500 CB	LEU 502 CD1	–0.127	3.887
	ARG 501 O	TYR 475 CB	–0.165	3.465
	ARG 501 NH2	SER 468 C	–0.191	3.441
	THR 499 CB	ASP 497 OD1	–0.194	3.494
	THR 499 N	ASP 497 OD1	–0.204	2.864
	ARG 501 CA	LEU 467 CD2	–0.207	3.967
	ARG 501 NH2	ASN 470 CB	–0.245	3.765
	ARG 501 NH1	ASN 470 ND2	–0.249	3.529
	GLU 498 N	ASP 497 OD1	–0.251	2.911
	ARG 501 CZ	SER 468 O	–0.252	3.282
	ARG 501 NH2	SER 468 N	–0.277	3.557
	THR 499 CA	ASP 497 OD1	–0.293	3.593
	ARG 501 C	ASP 497 O	–0.305	3.335
	ARG 501 O	TYR 475 CD1	–0.314	3.494

			GLU 498 CB	LEU 467 CD2	–0.321	4.081
			SER 500 N	ASP 497 CG	–0.353	3.873
			ARG 501 NH1	SER 468 CB	–0.354	3.874
			ARG 501 CZ	SER 468 OG	–0.372	3.442
			ARG 501 N	ASP 497 C	–0.396	3.646
			SER 500 O	LEU 502 CG	–0.396	3.696
			SER 500 O	TYR 475 CD1	–0.397	3.577
	T499	5	THR 499 OG1	ASP 497 CG	0.105	3.235
			THR 499 OG1	ASP 497 OD1	–0.068	2.548
			THR 499 CB	ASP 497 OD1	–0.194	3.494
			THR 499 N	ASP 497 OD1	–0.204	2.864
			THR 499 CA	ASP 497 OD1	–0.293	3.593
Vip3Aa–N499S	β14–β15 loop (E^{498}SSR^{501})	36	ARG 501 CA	ASP 497 O	0.464	2.836
			GLU 498 CD	THR 466 O	0.392	2.908
			ARG 501 NH1	ASN 470 CB	0.287	3.233
			ARG 501 NH2	SER 468 O	0.233	2.427
			ARG 501 CZ	ASN 470 CB	0.2	3.29
			GLU 498 CA	LEU 467 CD2	0.16	3.6
			ARG 501 NH1	SER 468 OG	0.132	2.568
			ARG 501 N	ASP 497 O	0.117	2.543
			SER 500 CB	LEU 502 CG	0.098	3.662
			GLU 498 OE1	THR 466 O	0.089	2.751
			ARG 501 O	VAL 474 CG1	0.044	3.256
			SER 499 OG	ASP 497 CG	0.042	3.298

GLU 498 CG	THR 466 O	–0.056	3.356
ARG 501 NE	ASN 470 CB	–0.102	3.622
SER 500 CB	LEU 502 CD1	–0.127	3.887
SER 499 OG	ASP 497 OD1	–0.155	2.635
ARG 501 O	TYR 475 CB	–0.165	3.465
SER 499 CB	ASP 497 OD1	–0.174	3.474
ARG 501 NH2	SER 468 C	–0.191	3.441
SER 499 N	ASP 497 OD1	–0.204	2.864
ARG 501 CA	LEU 467 CD2	–0.207	3.967
ARG 501 NH2	ASN 470 CB	–0.245	3.765
ARG 501 NH1	ASN 470 ND2	–0.249	3.529
GLU 498 N	ASP 497 OD1	–0.251	2.911
ARG 501 CZ	SER 468 O	–0.252	3.282
ARG 501 NH2	SER 468 N	–0.277	3.557
SER 499 CA	ASP 497 OD1	–0.293	3.593
ARG 501 C	ASP 497 O	–0.305	3.335
ARG 501 O	TYR 475 CD1	–0.314	3.494
GLU 498 CB	LEU 467 CD2	–0.321	4.081
SER 500 N	ASP 497 CG	–0.353	3.873
ARG 501 NH1	SER 468 CB	–0.354	3.874
ARG 501 CZ	SER 468 OG	–0.372	3.442
ARG 501 N	ASP 497 C	–0.396	3.646
SER 500 O	LEU 502 CG	–0.396	3.696
SER 500 O	TYR 475 CD1	–0.397	3.577

	S499	5	SER 499 OG	ASP 497 CG	0.042	3.298
			SER 499 OG	ASP 497 OD1	–0.155	2.635
			SER 499 CB	ASP 497 OD1	–0.174	3.474
			SER 499 N	ASP 497 OD1	–0.204	2.864
			SER 499 CA	ASP 497 OD1	–0.293	3.593
Vip3Aa–S500T	β14–β15 loop ($E^{498}NTR^{501}$)	42	THR 500 OG1	LEU 502 CG	0.505	2.835
			ARG 501 CA	ASP 497 O	0.464	2.836
			GLU 498 CD	THR 466 O	0.392	2.908
			THR 500 OG1	LEU 502 CD1	0.387	2.953
			ARG 501 NH1	ASN 470 CB	0.287	3.233
			ARG 501 NH2	SER 468 O	0.233	2.427
			ARG 501 CZ	ASN 470 CB	0.2	3.29
			GLU 498 CA	LEU 467 CD2	0.16	3.6
			ARG 501 NH1	SER 468 OG	0.132	2.568
			ARG 501 N	ASP 497 O	0.117	2.543
			THR 500 CB	LEU 502 CG	0.114	3.646
			GLU 498 OE1	THR 466 O	0.089	2.751
			ARG 501 O	VAL 474 CG1	0.044	3.256
			THR 500 OG1	ASP 497 OD2	0.039	2.441
			THR 500 CB	ASP 497 OD2	0.005	3.295
			THR 500 OG1	ASP 497 CG	0.004	3.336
			GLU 498 CG	THR 466 O	–0.056	3.356
			THR 500 N	ASP 497 OD2	–0.085	2.745
			ARG 501 NE	ASN 470 CB	–0.102	3.622

		THR 500 CB	LEU 502 CD1	–0.113	3.873
		ARG 501 O	TYR 475 CB	–0.165	3.465
		ARG 501 NH2	SER 468 C	–0.191	3.441
		THR 500 CA	ASP 497 OD2	–0.192	3.492
		ARG 501 CA	LEU 467 CD2	–0.207	3.967
		ARG 501 NH2	ASN 470 CB	–0.245	3.765
		ARG 501 NH1	ASN 470 ND2	–0.249	3.529
		ARG 501 CZ	SER 468 O	–0.252	3.282
		THR 500 CG2	ASP 497 OD2	–0.264	3.564
		ARG 501 NH2	SER 468 N	–0.277	3.557
		ARG 501 C	ASP 497 O	–0.305	3.335
		ARG 501 O	TYR 475 CD1	–0.314	3.494
		GLU 498 CB	LEU 467 CD2	–0.321	4.081
		THR 500 OG1	ASP 497 CB	–0.327	3.667
		THR 500 OG1	LEU 502 CB	–0.352	3.692
		THR 500 N	ASP 497 CG	–0.353	3.873
		ARG 501 NH1	SER 468 CB	–0.354	3.874
		ARG 501 CZ	SER 468 OG	–0.372	3.442
		ASN 499 O	THR 500 CG2	–0.372	3.672
		ASN 499 CB	ASP 497 OD2	–0.396	3.696
		ARG 501 N	ASP 497 C	–0.396	3.646
		THR 500 O	LEU 502 CG	–0.396	3.696
		THR 500 O	TYR 475 CD1	–0.397	3.577
	T500	16	THR 500 OG1	LEU 502 CG	0.505	2.835

			THR 500 OG1	LEU 502 CD1	0.387	2.953
			THR 500 CB	LEU 502 CG	0.114	3.646
			THR 500 OG1	ASP 497 OD2	0.039	2.441
			THR 500 CB	ASP 497 OD2	0.005	3.295
			THR 500 OG1	ASP 497 CG	0.004	3.336
			THR 500 N	ASP 497 OD2	–0.085	2.745
			THR 500 CB	LEU 502 CD1	–0.113	3.873
			THR 500 CA	ASP 497 OD2	–0.192	3.492
			THR 500 CG2	ASP 497 OD2	–0.264	3.564
			THR 500 OG1	ASP 497 CB	–0.327	3.667
			THR 500 OG1	LEU 502 CB	–0.352	3.692
			THR 500 N	ASP 497 CG	–0.353	3.873
			THR 500 CG2	ASN 499 O	–0.372	3.672
			THR 500 O	LEU 502 CG	–0.396	3.696
			THR 500 O	TYR 475 CD1	–0.397	3.577
Vip3Aa–S500N	β14–β15 loop ($E^{498}NNR^{501}$)	34	ARG 501 CA	ASP 497 O	0.464	2.836
			GLU 498 CD	THR 466 O	0.392	2.908
			ARG 501 NH1	ASN 470 CB	0.287	3.233
			ARG 501 NH2	SER 468 O	0.233	2.427
			ARG 501 CZ	ASN 470 CB	0.2	3.29
			GLU 498 CA	LEU 467 CD2	0.16	3.6
			ARG 501 NH1	SER 468 OG	0.132	2.568
			ARG 501 N	ASP 497 O	0.117	2.543
			GLU 498 OE1	THR 466 O	0.089	2.751

ASN 500 CB	LEU 502 CG	0.083	3.677
ARG 501 O	VAL 474 CG1	0.044	3.256
GLU 498 CG	THR 466 O	–0.056	3.356
ARG 501 NE	ASN 470 CB	–0.102	3.622
ASN 500 CB	LEU 502 CD1	–0.138	3.898
ARG 501 O	TYR 475 CB	–0.165	3.465
ASN 499 CB	ASP 497 OD1	–0.182	3.482
ARG 501 NH2	SER 468 C	–0.191	3.441
ASN 499 N	ASP 497 OD1	–0.204	2.864
ARG 501 CA	LEU 467 CD2	–0.207	3.967
ARG 501 NH2	ASN 470 CB	–0.245	3.765
ARG 501 NH1	ASN 470 ND2	–0.249	3.529
GLU 498 N	ASP 497 OD1	–0.251	2.911
ARG 501 CZ	SER 468 O	–0.252	3.282
ARG 501 NH2	SER 468 N	–0.277	3.557
ASN 499 CA	ASP 497 OD1	–0.293	3.593
ARG 501 C	ASP 497 O	–0.305	3.335
ARG 501 O	TYR 475 CD1	–0.314	3.494
GLU 498 CB	LEU 467 CD2	–0.321	4.081
ASN 500 N	ASP 497 CG	–0.353	3.873
ARG 501 NH1	SER 468 CB	–0.354	3.874
ARG 501 CZ	SER 468 OG	–0.372	3.442
ARG 501 N	ASP 497 C	–0.396	3.646
ASN 500 O	LEU 502 CG	–0.396	3.696

			ASN 500 O	TYR 475 CD1	–0.397	3.577
	N500	5	ASN 500 CB	LEU 502 CG	0.083	3.677
			ASN 500 CB	LEU 502 CD1	–0.138	3.898
			ASN 500 N	ASP 497 CG	–0.353	3.873
			ASN 500 O	LEU 502 CG	–0.396	3.696
			ASN 500 O	TYR 475 CD1	–0.397	3.577
Vip3Aa–R501A	*β*14–*β*15 loop ($E^{498}NSA^{501}$)	22	ALA 501 CA	ASP 497 O	0.464	2.836
			GLU 498 CD	THR 466 O	0.392	2.908
			GLU 498 CA	LEU 467 CD2	0.16	3.6
			ALA 501 N	ASP 497 O	0.117	2.543
			SER 500 CB	LEU 502 CG	0.098	3.662
			GLU 498 OE1	THR 466 O	0.089	2.751
			ALA 501 O	VAL 474 CG1	0.044	3.256
			GLU 498 CG	THR 466 O	–0.056	3.356
			SER 500 CB	LEU 502 CD1	–0.127	3.887
			ALA 501 O	TYR 475 CB	–0.165	3.465
			ASN 499 CB	ASP 497 OD1	–0.182	3.482
			ASN 499 N	ASP 497 OD1	–0.204	2.864
			ALA 501 CA	LEU 467 CD2	–0.207	3.967
			GLU 498 N	ASP 497 OD1	–0.251	2.911
			ASN 499 CA	ASP 497 OD1	–0.293	3.593
			ALA 501 C	ASP 497 O	–0.305	3.335
			ALA 501 O	TYR 475 CD1	–0.314	3.494
			GLU 498 CB	LEU 467 CD2	–0.321	4.081

			SER 500 N	ASP 497 CG	–0.353	3.873
			ALA 501 N	ASP 497 C	–0.396	3.646
			SER 500 O	LEU 502 CG	–0.396	3.696
			SER 500 O	TYR 475 CD1	–0.397	3.577
	A501	8	ALA 501 CA	ASP 497 O	0.464	2.836
			ALA 501 N	ASP 497 O	0.117	2.543
			ALA 501 O	VAL 474 CG1	0.044	3.256
			ALA 501 O	TYR 475 CB	–0.165	3.465
			ALA 501 CA	LEU 467 CD2	–0.207	3.967
			ALA 501 C	ASP 497 O	–0.305	3.335
			ALA 501 O	TYR 475 CD1	–0.314	3.494
			ALA 501 N	ASP 497 C	–0.396	3.646
Vip3Aa	*β*12–*β*13 loop (S^{468}ANDDG^{473})	52	GLY 473 CA	VAL 338 O	0.271	2.909
			ASP 471 O	LYS 339 CE	0.142	3.038
			ALA 469 HB1	GLN 404 O	0.114	2.366
			ASP 472 OD1	ASP 471 CG	0.081	3.099
			ASP 471 O	LYS 339 HE2	0.055	2.425
			ALA 469 CB	GLN 404 O	0.034	3.146
			ASN 470 OD1	ASP 472 HB2	0.034	2.446
			GLY 473 HA3	VAL 338 O	0.032	2.448
			ALA 469 CB	GLU 406 H	0.017	2.683
			ASN 470 OD1	ASP 472 CB	–0.002	3.182
			ALA 469 HB2	GLU 406 N	–0.025	2.65
			ALA 469 CA	GLU 406 H	–0.027	2.727

	ASP 472 OD1	ASP 471 OD2	–0.037	2.997
	ASP 472 H	ASN 470 OD1	–0.06	2.14
	ASP 472 H	ASP 471 CG	–0.062	2.762
	ALA 469 CB	GLU 406 N	–0.073	3.398
	ASP 472 N	ASN 470 OD1	–0.081	2.786
	ALA 469 HB2	GLU 406 H	–0.082	2.082
	ASP 471 O	LYS 339 NZ	–0.089	2.794
	ASP 472 N	ASP 471 OD1	–0.127	2.832
	ASP 471 OD1	ASP 472 OD1	–0.132	3.092
	ASP 472 H	ASP 471 OD1	–0.142	2.222
	ASN 470 OD1	ASP 472 CA	–0.148	3.328
	GLY 473 C	VAL 338 O	–0.152	3.332
	GLY 473 N	LYS 339 HE2	–0.161	2.786
	ALA 469 HA	GLU 406 H	–0.177	2.177
	ALA 469 CA	GLU 406 N	–0.187	3.512
	ALA 469 N	GLU 406 HG2	–0.193	2.818
	ALA 469 H	GLU 406 HG2	–0.194	2.194
	ASP 472 C	LYS 339 HE2	–0.195	2.895
	GLY 473 HA2	VAL 338 O	–0.198	2.678
	GLY 473 N	LYS 339 CE	–0.213	3.538
	ASN 470 CG	ASP 471 H	–0.259	2.959
	GLY 473 CA	LYS 339 CG	–0.269	3.669
	ALA 469 N	GLU 406 CG	–0.27	3.595
	ASP 472 HB3	LYS 337 HZ3	–0.278	2.278

		ALA 469 N	GLU 406 HA	–0.282	2.907
		ASN 470 CG	ASP 472 H	–0.295	2.995
		ALA 469 H	GLU 406 CG	–0.3	3
		ASN 470 CG	ASP 471 OD1	–0.302	3.482
		ASP 471 OD1	ASN 470 OD1	–0.303	3.263
		ASN 470 O	ASP 472 O	–0.305	3.265
		GLY 473 CA	LYS 339 HG2	–0.325	3.025
		ASP 471 O	LYS 339 HZ2	–0.325	2.405
		ASN 470 CG	ASP 472 N	–0.346	3.671
		ALA 469 N	GLU 406 CA	–0.348	3.673
		ASP 472 O	ASN 470 OD1	–0.357	3.317
		ALA 469 HA	GLU 406 N	–0.361	2.986
		GLY 473 HA2	LYS 339 HG2	–0.367	2.367
		ASP 472 O	ASP 471 O	–0.378	3.338
		ASP 471 C	ASP 472 OD1	–0.378	3.558
		ASP 472 CB	LYS 337 HZ3	–0.39	3.09
	N470	12	ASN 470 OD1	ASP 472 HB2	0.034	2.446
			ASN 470 OD1	ASP 472 CB	–0.002	3.182
			ASN 470 OD1	ASP 472 H	–0.06	2.14
			ASN 470 OD1	ASP 472 N	–0.081	2.786
			ASN 470 OD1	ASP 472 CA	–0.148	3.328
			ASN 470 CG	ASP 471 H	–0.259	2.959
			ASN 470 CG	ASP 472 H	–0.295	2.995
			ASN 470 CG	ASP 471 OD1	–0.302	3.482

			ASN 470 OD1	ASP 471 OD1	–0.303	3.263
			ASN 470 O	ASP 472 O	–0.305	3.265
			ASN 470 CG	ASP 472 N	–0.346	3.671
			ASN 470 OD1	ASP 472 O	–0.357	3.317
Vip3Aa–N470K	β12–β13 loop (S^{468}AKDDG^{473})	39	ASP 471 O	LYS 339 CE	0.622	2.678
			GLY 473 N	LYS 337 CE	0.457	3.063
			ASP 472 CB	LYS 337 CE	0.306	3.454
			ASP 471 O	LYS 339 NZ	0.294	2.366
			ASP 472 CG	LYS 337 NZ	0.292	3.228
			ASP 472 CB	LYS 337 NZ	0.257	3.263
			ALA 469 CB	GLU 406 N	0.205	3.315
			GLY 473 CA	VAL 338 O	0.185	3.115
			SER 468 O	ARG 501 NH2	0.176	2.484
			LYS 470 CD	ARG 501 CD	0.174	3.586
			ALA 469 N	GLU 406 CG	0.164	3.356
			GLY 473 CA	LYS 339 CG	0.13	3.63
			GLY 473 N	LYS 339 CE	0.019	3.501
			ALA 469 CB	GLU 406 CG	0.009	3.751
			ALA 469 CA	GLU 406 N	–0.021	3.541
			GLY 473 CA	LYS 337 CG	–0.052	3.812
			GLY 473 CA	LYS 337 CE	–0.084	3.844
			ASP 472 CA	LYS 337 CE	–0.142	3.902
			ALA 469 CA	GLU 406 CA	–0.151	3.911
			ASP 472 C	LYS 339 CE	–0.164	3.654

		ALA 469 N	GLU 406 CA	−0.177	3.697
		ALA 469 CB	SER 405 CA	−0.188	3.948
		GLY 473 CA	LYS 339 CE	−0.198	3.958
		LYS 470 O	ASP 472 O	−0.198	3.038
		SER 468 O	ARG 501 CZ	−0.2	3.23
		ASP 472 CA	LYS 339 CE	−0.201	3.961
		LYS 470 CG	ASP 472 N	−0.234	3.754
		ASP 472 CG	LYS 337 CE	−0.244	4.004
		LYS 470 CE	ASP 472 CB	−0.261	4.021
		LYS 470 CB	ARG 501 CZ	−0.264	3.754
		ALA 469 CA	GLU 406 CG	−0.276	4.036
		LYS 470 CB	ARG 501 NH2	−0.288	3.808
		SER 468 C	ARG 501 NH2	−0.292	3.542
		ALA 469 CB	GLU 406 CA	−0.303	4.063
		ASP 471 C	LYS 339 NZ	−0.306	3.556
		ASP 471 C	LYS 339 CE	−0.345	3.835
		GLY 473 C	LYS 337 CG	−0.37	3.86
		SER 468 N	ARG 501 NH2	−0.374	3.654
		SER 468 CA	GLU 406 CG	−0.375	4.135
K470	6	LYS 470 CD	ARG 501 CD	0.174	3.586
		LYS 470 O	ASP 472 O	−0.198	3.038
		LYS 470 CG	ASP 472 N	−0.234	3.754
		LYS 470 CE	ASP 472 CB	−0.261	4.021
		LYS 470 CB	ARG 501 CZ	−0.264	3.754

	LYS 470 CB	ARG 501 NH2	–0.288	3.808
	LYS 470 CD	ARG 501 CD	0.174	3.586

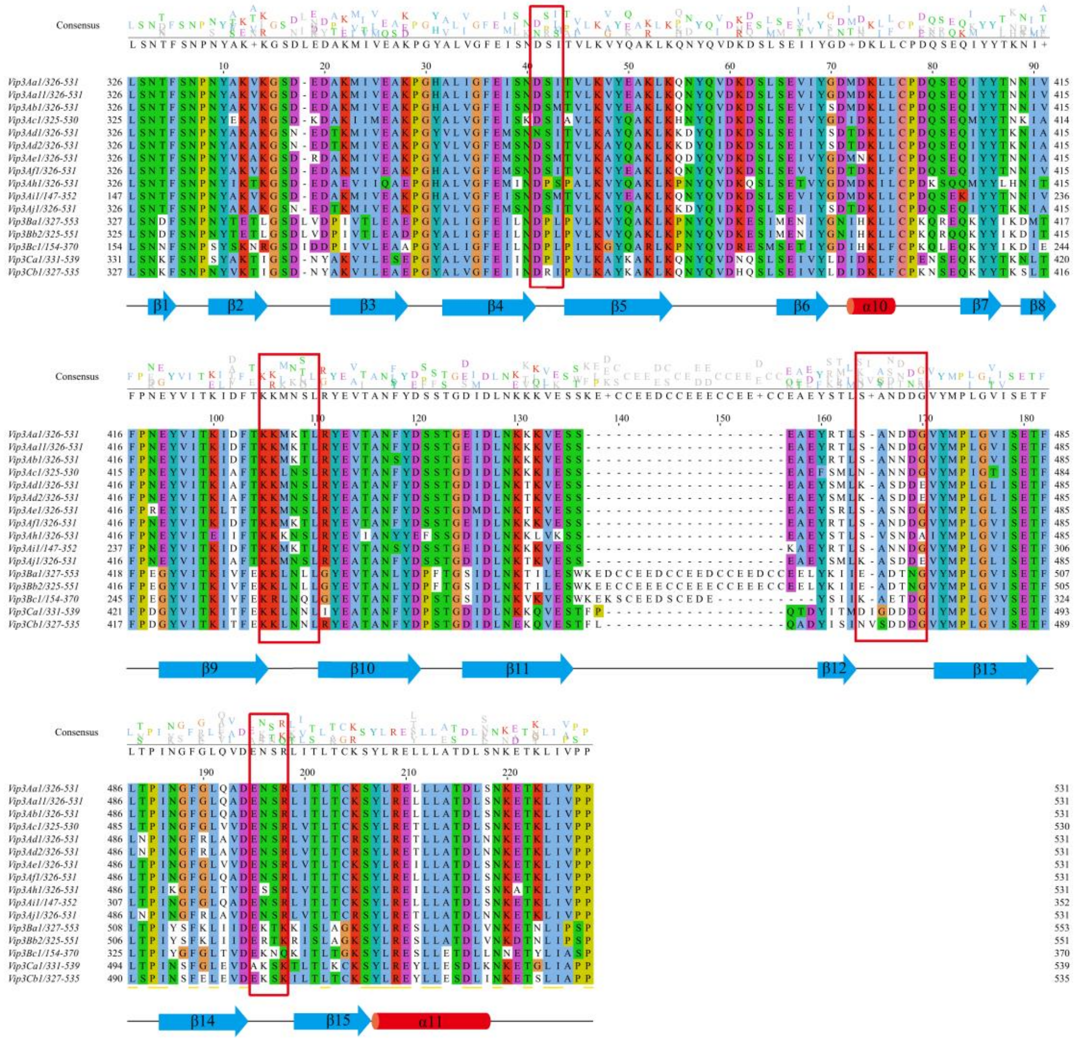

Figure S1. Alignment of the amino acid sequences in domain III. The accession number of Vip3Aa1, Vip3Aa11, Vip3Ab1, Vip3Ac1, Vip3Ad1, Vip3Ad2, Vip3Ae1, Vip3Af1, Vip3Ah1, Vip3Ai1, Vip3Aj1, Vip3Ba1, Vip3Bb2, Vip3Bc1, Vip3Ca1, Vip3Cb1 was AAC37036, AAR36859, AAR40284, ABL23218, ABL23219, CAI43276, CAI43277, CAI43275, ABH10614, AGU13858, AIT93172, AAV70653, ABO30520, ATD53733, ADZ46178, LQ835767, respectively.

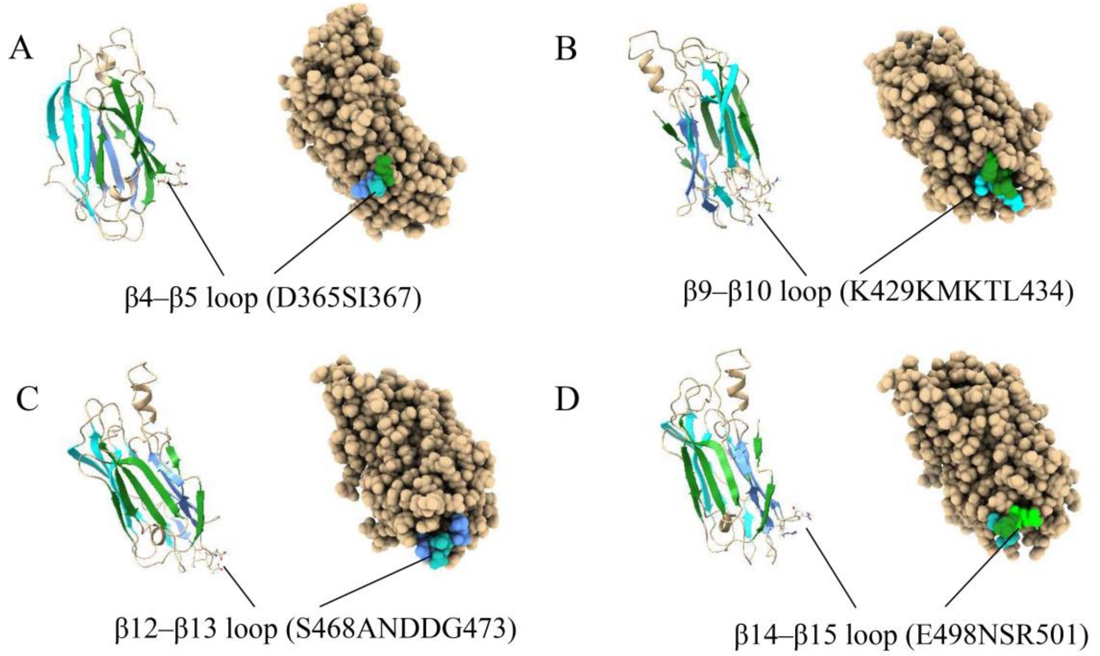

Figure S2. The four special loops exposed on the surface of the Vip3Aa protein domain III. (A) *β*4–*β*5 loop. (B) *β*9–*β*10 loop. (C) *β*12–*β*13 loop. (D) *β*14–*β*15 loop.

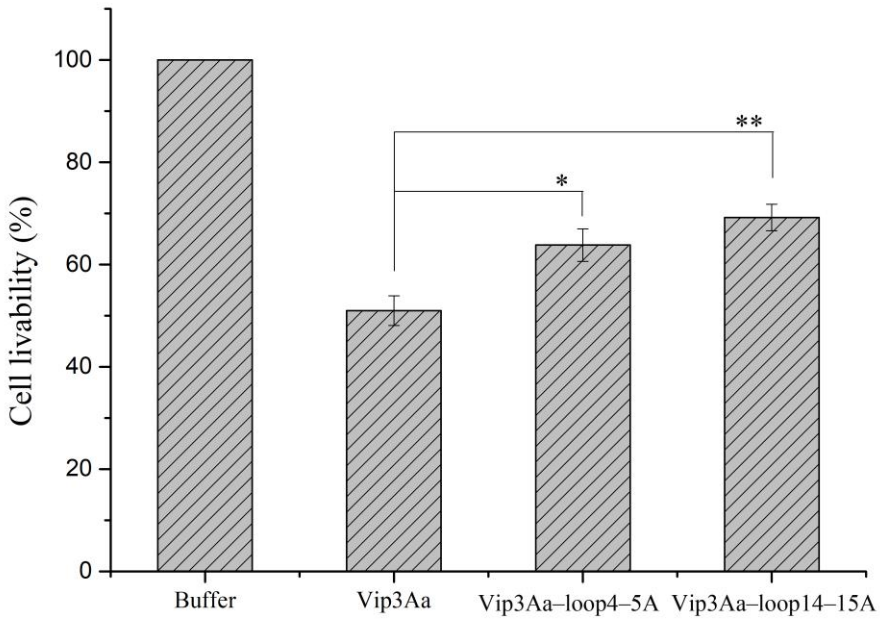

Figure S3. Effect of Vip3Aa, Vip3Aa–loop4–5A, and Vip3Aa–loop14–15A the cell livability of Sf9 cells. The final concentration of Vip3Aa or mutants used in the Cell Viability Assay was 40 μg/mL. The cell viability was detected using the CCK–8 Counting Kit (Dojindo, Kumamoto, Japan).

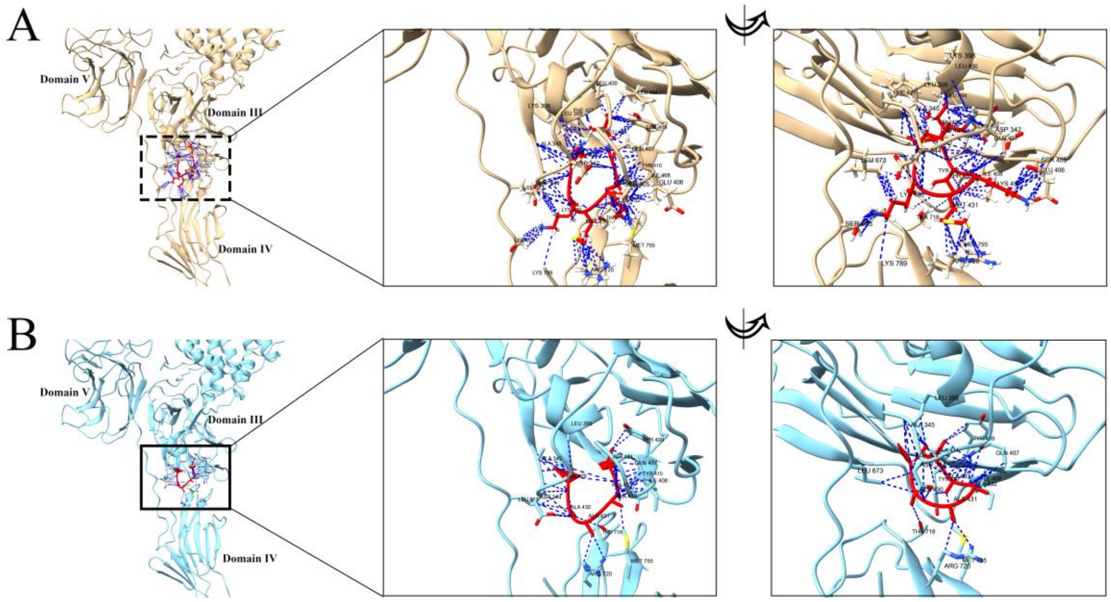

Figure S4. Contacts analysis between residues by chimera X. (A) Vip3Aa. (B) Vip3Aa–loop 9–10A.

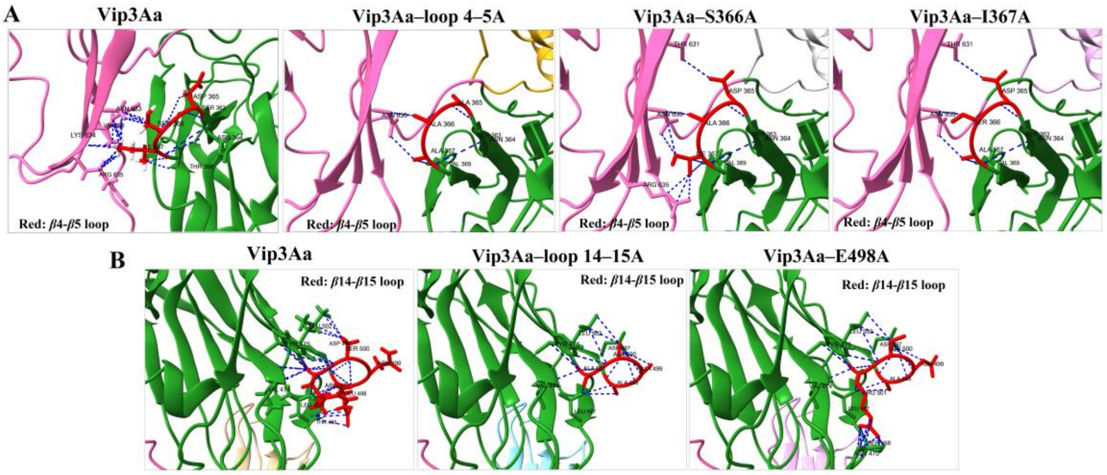

Figure S5. Contacts analysis between residues by chimera X. (A) Vip3Aa and β4–β5 loop mutants. (B) Vip3Aa and β14–β15 loop mutants. Green: domain III, pink: domain IV.

Table S3. Contact residues analysis with residues in β9–β10 loop.

Allowed overlap: –0.4

H–bond overlap reduction: 0.4

Ignore contacts between atoms separated by 4 bonds or less

Detect intra–residue contacts: False

Vip3Aa: 156 contacts

atom1	atom2	overlap	distance	atom1	atom2	overlap	distance
LEU 434 H	ILE 408 O	0.272	1.808	LYS 429 CA	ALA 345 O	–0.186	3.366
LYS 429 O	ASP 344 OD1	0.246	2.714	LYS 430 O	ASP 344 CG	–0.187	3.367
LEU 434 N	ILE 408 O	0.237	2.468	THR 433 CG2	MET 755 HE3	–0.187	2.887
THR 433 CG2	MET 755 CE	0.181	3.219	LYS 429 N	ALA 345 O	–0.188	2.893
LYS 430 NZ	SER 675 CB	0.153	3.172	LEU 434 H	ILE 408 C	–0.189	2.889
LEU 434 CD2	TYR 409 HD1	0.143	2.557	LYS 429 HZ1	ASP 342 HA	–0.189	2.189
THR 433 O	LYS 429 HA	0.138	2.342	LYS 429 NZ	ASP 342 CA	–0.19	3.515
LYS 432 O	ILE 408 N	0.108	2.597	LYS 429 O	ASP 344 C	–0.198	3.378
LYS 429 O	ASP 344 HA	0.094	2.386	LYS 429 CE	GLN 407 OE1	–0.198	3.378
LYS 432 O	ILE 408 H	0.093	1.987	LYS 432 O	GLN 407 CD	–0.2	3.38
LEU 434 CD2	TYR 409 CD1	0.091	3.309	LYS 432 HZ3	SER 405 HB2	–0.2	2.2
LYS 430 HZ1	SER 675 CB	0.076	2.624	LEU 434 HD21	LEU 400 CD2	–0.207	2.907
LYS 429 HB3	ALA 345 O	0.064	2.416	MET 431 CE	ARG 720 NE	–0.211	3.536
THR 433 HA	ILE 408 O	0.062	2.418	MET 431 HE2	ARG 720 NH2	–0.214	2.839
LYS 432 NZ	SER 405 HB2	0.053	2.572	THR 433 HG21	THR 718 HG21	–0.222	2.222
LEU 434 CB	TYR 409 CD1	–0.058	3.458	MET 431 HE2	ARG 720 CD	–0.223	2.923

LYS 432 HE2	SER 405 OG	–0.062	2.562	LYS 430 HA	LEU 673 CD2	–0.225	2.925
LYS 429 CE	GLN 407 HE22	–0.066	2.766	MET 431 HE2	ARG 720 HD2	–0.227	2.227
LYS 432 O	GLN 407 CA	–0.067	3.247	LYS 429 HD2	LEU 399 O	–0.228	2.708
THR 433 HG21	MET 755 CE	–0.071	2.771	THR 433 CA	ILE 408 HB	–0.232	2.932
LYS 429 HB2	LEU 399 CD2	–0.078	2.778	LYS 430 HZ2	SER 675 CB	–0.234	2.934
MET 431 CE	ARG 720 HD2	–0.089	2.789	LEU 434 CB	ILE 408 O	–0.238	3.418
LYS 432 O	GLN 407 C	–0.092	3.272	LEU 434 HD22	TYR 409 CD1	–0.239	2.939
LYS 429 HE3	GLN 407 HE22	–0.098	2.098	LYS 430 O	ASP 344 CB	–0.244	3.424
LYS 429 O	ASP 344 CG	–0.099	3.279	LYS 432 H	THR 433 H	–0.245	2.245
LEU 434 CD1	LEU 399 HD22	–0.103	2.803	LEU 434 HD22	TYR 409 HE1	–0.248	2.248
LEU 434 CB	TYR 409 HD1	–0.106	2.806	LYS 432 CE	SER 405 OG	–0.251	3.451
LYS 429 HE3	GLN 407 NE2	–0.109	2.734	LEU 434 CA	ILE 408 O	–0.26	3.44
LEU 434 HB3	TYR 409 CD1	–0.11	2.81	LYS 430 CE	LYS 789 O	–0.261	3.441
THR 433 HA	ILE 408 HB	–0.114	2.114	LYS 429 HE3	GLN 407 CD	–0.267	2.967
LYS 429 CE	GLN 407 NE2	–0.119	3.444	THR 433 HA	ILE 408 CB	–0.269	2.969
MET 431 SD	ARG 720 HD2	–0.13	2.912	LYS 429 HB3	ALA 345 N	–0.269	2.894
LYS 432 NZ	SER 405 CB	–0.135	3.46	THR 433 HG21	MET 755 HE3	–0.272	2.272
LYS 429 CD	GLN 407 HE22	–0.139	2.839	LYS 430 HZ1	SER 675 HB3	–0.275	2.275
LYS 429 O	LYS 430 O	–0.14	3.1	THR 433 HG22	ILE 408 CB	–0.278	2.978
LYS 430 HZ2	SER 675 HB3	–0.141	2.141	MET 431 HB2	LYS 432 H	–0.28	2.28
LYS 432 HD3	SER 405 O	–0.143	2.623	THR 433 HG22	ILE 408 HD12	–0.283	2.283
MET 431 O	LYS 432 CG	–0.144	3.324	LYS 429 HB3	ALA 345 C	–0.283	2.983
LEU 434 HD11	PHE 427 HD2	–0.144	2.144	MET 431 SD	ARG 720 HD3	–0.294	3.076
LEU 434 HB3	TYR 409 HD1	–0.145	2.145	LYS 430 HZ1	SER 675 HB2	–0.295	2.295

MET 431 C	LYS 432 HG3	–0.151	2.851	LEU 434 CD1	LEU 399 CD2	–0.296	3.696
LEU 434 CD1	PHE 427 HD2	–0.152	2.852	LYS 432 CE	SER 405 O	–0.297	3.477
LEU 434 CD2	TYR 409 CE1	–0.156	3.556	LEU 434 HB2	GLN 407 HE21	–0.298	2.298
LYS 432 HE2	SER 405 HG	–0.159	2.159	LYS 429 HZ1	ASP 342 CA	–0.299	2.999
LEU 434 HD22	TYR 409 CE1	–0.164	2.864	LYS 430 O	ASP 344 CA	–0.299	3.479
LYS 430 HG3	MET 431 N	–0.167	2.792	LYS 430 O	ASP 344 HA	–0.3	2.78
LYS 432 CD	SER 405 O	–0.171	3.351	LYS 429 CD	GLN 407 NE2	–0.301	3.626
LEU 434 HD23	TYR 409 CD1	–0.171	2.871	LYS 430 HD3	LEU 673 CD2	–0.301	3.001
THR 433 O	LYS 429 CG	–0.173	3.353	LYS 430 CD	LEU 673 HD21	–0.302	3.002
LYS 429 HB2	LEU 399 HD23	–0.176	2.176	THR 433 CG2	ILE 408 HB	–0.305	3.005
THR 433 HG22	ILE 408 HB	–0.181	2.181	LEU 434 N	ILE 408 C	–0.311	3.636
LYS 432 HZ1	SER 405 HB2	–0.312	2.312	LEU 434 CD2	TYR 409 HE1	–0.368	3.068
LYS 432 O	GLN 407 OE1	–0.314	3.274	MET 431 HE2	ARG 720 CZ	–0.37	3.07
LYS 430 HD2	LEU 673 CD2	–0.314	3.014	LYS 429 C	ALA 345 O	–0.375	3.555
LYS 430 CD	LEU 673 HD23	–0.318	3.018	LYS 432 CE	SER 405 HG	–0.376	3.076
LEU 434 HD23	LEU 494 CD1	–0.32	3.02	LEU 434 O	TYR 410 H	–0.378	2.458
MET 431 CE	ARG 720 CZ	–0.33	3.73	LEU 434 CD1	PHE 427 CD2	–0.381	3.781
LYS 432 O	ILE 408 CA	–0.33	3.51	THR 433 HA	ILE 408 C	–0.385	3.085
LYS 432 HD3	GLU 406 HB2	–0.331	2.331	LYS 429 CE	GLN 407 CD	–0.387	3.787
LYS 432 O	ILE 408 CB	–0.331	3.511	MET 431 HG3	ARG 720 HD2	–0.39	2.39
LYS 429 NZ	LYS 398 O	–0.336	3.041	LYS 429 HZ3	LYS 398 O	–0.393	2.473
LYS 429 HZ3	ASP 342 HA	–0.338	2.338	LEU 434 CD2	LEU 494 CD1	–0.393	3.793
LYS 430 HD3	LEU 673 HD21	–0.338	2.338	THR 433 CG2	ILE 408 HD12	–0.395	3.095
LYS 432 CE	SER 405 CB	–0.353	3.753	THR 433 HG22	ILE 408 CD1	–0.396	3.096

MET 431 CG	ARG 720 HD2	–0.354	3.054	MET 431 CE	ARG 720 NH2	–0.397	3.722
LEU 434 HD11	PHE 427 CD2	–0.354	3.054	LEU 434 HD22	TYR 409 HD1	–0.397	2.397
LEU 434 CD2	LEU 400 CD2	–0.359	3.759	LYS 430 NZ	SER 675 HB2	–0.398	3.023
LYS 430 HD2	LEU 673 HD23	–0.361	2.361	LYS 432 HE2	SER 405 CB	–0.399	3.099

Vip3Aa–loop9–10A: 47 contacts

atom1	atom2	overlap	distance	atom1	atom2	overlap	distance
ALA 430 O	ASP 344 OD1	0.967	1.873	ALA 433 CA	GLN 407 NE2	–0.115	3.635
ALA 430 O	ASP 344 CG	0.676	2.624	ALA 433 C	ILE 408 O	–0.134	3.164
ALA 434 CB	TYR 409 CD2	0.578	3.062	ALA 434 CB	ILE 408 O	–0.141	3.441
ALA 431 CB	ARG 720 CD	0.338	3.422	ALA 433 C	GLN 407 NE2	–0.146	3.396
ALA 431 CB	ARG 720 NH2	0.306	3.214	ALA 429 CB	ALA 345 N	–0.186	3.706
ALA 429 CB	LEU 399 CD1	0.294	3.466	ALA 429 O	ALA 345 O	–0.204	3.044
ALA 434 CB	TYR 409 CE2	0.28	3.36	ALA 430 O	ASP 344 CB	–0.206	3.506
ALA 432 C	GLN 407 NE2	0.187	3.063	ALA 434 CA	ILE 408 O	–0.216	3.516
ALA 432 O	GLN 407 NE2	0.171	2.489	ALA 429 N	ALA 345 O	–0.225	2.885
ALA 433 CA	ILE 408 O	0.17	3.13	ALA 432 O	GLN 407 C	–0.272	3.302
ALA 432 O	ILE 408 N	0.153	2.507	ALA 433 CB	MET 755 CE	–0.272	4.032
ALA 434 N	ILE 408 O	0.137	2.523	ALA 433 N	GLN 407 NE2	–0.274	3.554
ALA 429 CB	ALA 345 O	0.122	3.178	ALA 430 O	ASP 344 CA	–0.316	3.616
ALA 429 O	ASP 344 CA	0.12	3.18	ALA 434 N	GLN 407 NE2	–0.319	3.599
ALA 429 CA	ALA 433 O	0.067	3.233	ALA 429 O	LEU 673 CD2	–0.325	3.625
ALA 433 CB	TYR 410 CE1	–0.011	3.651	ALA 430 C	ASP 344 CG	–0.331	3.821
ALA 430 CA	LEU 673 CD2	–0.038	3.798	ALA 429 CB	ALA 345 C	–0.36	3.85
ALA 432 O	ILE 408 CA	–0.053	3.353	ALA 429 CB	ALA 345 CA	–0.369	4.129

ALA 433 CA	ILE 408 CB	–0.064	3.824	ALA 429 O	ASP 344 C	–0.378	3.408
ALA 429 CA	ALA 345 O	–0.064	3.364	ALA 434 O	TYR 410 CD1	–0.379	3.559
ALA 432 O	GLN 407 CA	–0.067	3.367	ALA 429 O	ALA 430 O	–0.38	3.22
ALA 430 C	ASP 344 OD1	–0.069	3.099	ALA 433 O	ALA 429 CB	–0.396	3.696
ALA 432 O	ILE 408 CB	–0.08	3.38	ALA 430 CB	THR 718 OG1	–0.399	3.739
ALA 432 O	GLN 407 CD	–0.104	3.134				
